# Racial and Ethnic Disparities in Pediatric Counseling on Nutrition, Lifestyle, and Weight: A Secondary Analysis of the BP-CATCH Randomized Clinical Trial

**DOI:** 10.1001/jamanetworkopen.2024.56238

**Published:** 2025-01-29

**Authors:** Moonseong Heo, Corinna J. Rea, Tammy M. Brady, David G. Bundy, Ezinne Sylvia Melikam, Kelly Orringer, Beth A. Tarini, Kimberly Giuliano, Katherine Twombley, Beatrice Goilav, Peterkaye Kelly, Myles S. Faith, Angelo Pietrobelli, Michael L. Rinke

**Affiliations:** 1 Department of Public Health Sciences, College of Social, Behavioral and Public Health Sciences, Clemson University, Clemson, South Carolina; 2 Department of Pediatrics, Boston Children’s Hospital and Harvard Medical School, Boston, Massachusetts; 3 Department of Pediatrics, Johns Hopkins University School of Medicine, Baltimore, Maryland; 4 Department of Pediatrics, Medical University of South Carolina, Charleston; 5 Division of General Pediatrics, Michigan Medicine, Ann Arbor; 6 Center for Translational Research, Children’s National Hospital, Washington, DC; 7 Department of Pediatrics, George Washington University, Washington, DC; 8 Department of Primary Care Pediatrics, Cleveland Clinic, Cleveland, Ohio; 9 Department of Pediatrics, The Children’s Hospital at Montefiore, Albert Einstein College of Medicine, Bronx, New York; 10 Department of Counseling, School and Educational Psychology, University at Buffalo–The State University of New York; 11 Pediatric Unit, Department of Surgical Sciences, Dentistry, Gynecology and Pediatrics, University of Verona, Verona, Italy; 12 Pennington Biomedical Research Center, Baton Rouge, Louisiana

## Abstract

**Importance:**

Pediatric obesity and hypertension are highly correlated. To mitigate both conditions, provision of counseling on nutrition, lifestyle, and weight to children with high blood pressure (BP) measurements is recommended.

**Objective:**

To examine racial and ethnic disparities in receipt of nutrition, lifestyle, and weight counseling among patients with high BP at pediatric primary care visits stratified by patients’ weight status.

**Design, Setting, and Participants:**

This was a post hoc secondary analysis of the BP-CATCH study, a matched, stepped-wedge cluster randomized clinical trial investigating the best methods to screen children with high BP measurements and manage their care. Urban, suburban, and rural pediatric primary care practices across the US with a multidisciplinary team of at least 1 physician, 1 nurse and another practice associate, and a hypertension specialist for their practice group submitted baseline data from clinical encounters documented between November 2018 and January 2019. Practices identified the first 17 eligible patients with high BP measurements each month. This analysis was conducted from October 2023 to July 2024.

**Exposures:**

Race and ethnicity (Black, Hispanic, White, and other [Asian, multiracial, other races, and unknown race]) and weight status (with or without obesity).

**Main Outcomes and Measures:**

Primary outcomes were receipt of counseling on nutrition, lifestyle, and weight during primary care visits. Baseline measures extracted from medical records included demographics, anthropometric measures, and systolic and diastolic BP.

**Results:**

Of 2677 participants from 59 practices, 1516 (56.6%) were male; mean (SD) age was 10.8 (5.2) years. A total of 593 (21.1%) were Black; 414 (15.5%), Hispanic; 1111 (41.5%), White; and 559 (20.9%), other race and ethnicity. The overall crude unadjusted rates of receiving counseling were 63.5% (n = 1564 of 2463) for nutrition, 57.6% (n = 1419 of 2462) for lifestyle, 47.5% (n = 571 of 1202) for weight, and 46.4% (n = 1142 of 2461) for all counseling topics. Compared with the other 3 groups, Hispanic participants received significantly higher adjusted rates of nutrition (78.6%; 95% CI, 73.5%-83.8%), lifestyle (69.3%; 95% CI, 63.6%-74.9%), and all 3 (52.1%; 95% CI, 46.1%-58.2%) counseling topics. There were no significant differences in rates of receiving weight counseling between any pairs of groups. These findings were consistent in general among participants without obesity, and no significant pairwise differences were noted among participants with obesity except that nutrition counseling rates were significantly different between White participants and those reporting other race and ethnicity (68.3% [95% CI, 61.1%-75.4%] vs 81.6% [95% CI, 74.2%-89.1%]; Bonferroni-corrected *P* = .02).

**Conclusions and Relevance:**

This secondary analysis of the BP-CATCH trial found that among children with high BP measurements, racial and ethnic disparities in receiving nutrition, lifestyle, and all 3 counseling topics were significant, although no significant disparities in receipt of weight counseling were noted. Racial disparities in receipt of counseling were not observed in participants with and without obesity.

**Trial Registration:**

ClinicalTrials.gov Identifier: NCT03783650

## Key Points

**Question:**

Are there racial and ethnic disparities in receipt of nutrition, lifestyle, and weight counseling among children with high blood pressure (BP) in US pediatric primary care settings?

**Findings:**

In this secondary analysis of baseline data from 2677 pediatric patients with high BP in the BP-CATCH trial, the rate of receiving counseling for all topics was 46%. Rates differed between participants with and without obesity, across race and ethnicity categories, and across counseling topics.

**Meaning:**

The findings suggest that identification of drivers of and barriers to counseling for pediatric patients with obesity and high BP to minimize inequities in care and optimize health outcomes is needed.

## Teaser

This secondary analysis of the BP-CATCH trial investigates racial and ethnic disparities in receipt of nutrition, lifestyle, and weight counseling during pediatric primary care visits among children with high blood pressure.

## Introduction

Childhood obesity is prevalent, often originates early in life, and disproportionately affects children from racial and ethnic minority groups and disadvantaged backgrounds.^1^ Pediatric obesity is a significant public health concern, and its health consequences not only affect childhood but also are carried into adulthood.^2,3^ Obesity-related health consequences encompass a broad range of comorbidities, including high blood pressure (BP), elevated cholesterol levels, type 2 diabetes, asthma, sleep apnea, joint problems, and mental health concerns.^4,5,6,7^ Despite such undesirable consequences, the US Centers for Disease Control and Prevention (CDC) reported that the prevalence of pediatric obesity has remained persistently high at 19.7% (ie, 1 in 5; n = 14.7 million) among children and adolescents aged 2 to 19 years in the US from 2017 to 2020.^8^ The prevalence varies by age, such that it rises with increasing age during childhood and adolescence, and by race and ethnicity, with the highest prevalence (26.2%) among Hispanic children.^8^

Pediatric hypertension, a significant correlate of obesity, also affects approximately 4.0% of children and adolescents in the US.^9,10^ The prevalence of elevated BP (previously known as prehypertension) ranges between 3% and 15%, and some argue that those prevalence values are underestimated.^11,12^ The unfavorable effects of pediatric hypertension^13^ are also carried into adulthood and are associated with adult hypertension, vascular diseases, and metabolic syndromes.^7,14,15^ Pediatric obesity and hypertension are associated with each other^16,17^: the prevalence of hypertension among children with obesity ranges from 5.6% to 18.3%, higher than that among groups with other weight status,^9,18,19^ and the prevalence of obesity among children with hypertension (31.9%) is also higher than that among children with BP in the reference range (11.5%) or elevated BP (24.6%).^18^ In addition, the extent of this association varies by race.^20,21,22^

Behavior interventions to promote increased physical activity and healthy diets are most often suggested to meet both obesity and hypertension public health challenges and have been associated with improved health outcomes among pediatric populations,^23,24,25,26,27,28,29^ if only marginally in some studies.^30^ Counseling or education centered around healthy lifestyle behaviors has been shown to have positive outcomes for children’s health status and to facilitate the development of skills to improve and maintain health as children mature into adulthood.^31^ As such, the American Academy of Pediatrics (AAP) evidence-based Clinical Practice Guidelines^32,33^ recommend behavior modification interventions, such as counseling on weight, lifestyle, and nutrition, during pediatric primary care visits, as these 3 areas are of critical importance to mitigating weight and high BP problems in children and adolescents.

Despite the AAP recommendation, counseling guidelines, and other obesity and weight control interventions for the pediatric population, racial and ethnic disparities persist in the prevalence of hypertension and obesity among pediatric patients.^8^ Furthermore, a limited number of studies have reported racial and ethnic disparities in receipt of recommended counseling among pediatric patients with high BP measurements in pediatric primary care settings. If such a counseling disparity exists, it may further broaden health care equity gaps in pediatric populations with obesity or hypertension. The present study aimed to examine and compare rates of receiving nutrition, lifestyle, and weight counseling during primary care visits in a pediatric population with at least 1 high BP measurement. Stratified analyses by race and ethnicity and weight groups were conducted using baseline data collected from the Boosting Primary Care Awareness and Treatment of Hypertension (BP-CATCH) quality improvement collaborative (QIC) study, which enrolled pediatric practices across the US.^34^

## Methods

### Design and Setting

The present study was a post hoc secondary analysis of baseline data collected for the BP-CATCH study, which was a prospective, matched, stepped-wedge cluster randomized clinical trial to investigate the best methods to screen children with high BP measurements and manage their care (NCT03783650). The BP-CATCH trial was designed to test whether a QIC reliably improved primary and specialty practitioner adherence to guideline-compliant care recommended by the AAP in 2017.^32^ The BP-CATCH trial enrolled primary care practice sites that identified a multidisciplinary team consisting of at least 1 physician, 1 nurse and another practice associate, and a hypertension specialist for their entire practice group. Each practice selected 1 project champion to lead on-site process improvement efforts as well as a data support person to conduct medical record reviews and enter data into REDCap. The BP-CATCH trial was collectively approved by the institutional review board (IRB) of the Albert Einstein College of Medicine, Bronx, New York, in addition to local IRBs when required. IRB approval for this secondary analysis was not required because the study followed the internal BP-CATCH publication policies. Given that the BP-CATCH trial was a quality improvement study, the Albert Eistein College of Medicine IRB determined that participant informed consent was not required. The reporting of the study results adhered to the Consolidated Standards of Reporting Trials (CONSORT) reporting guideline. The trial protocol is given in Supplement 1, and the CONSORT diagram for BP-CATCH is provided in the eFigure in Supplement 2.

### Participating Practices and Patients

Urban, suburban, and rural pediatric primary care practices from across the US were recruited for participation in the BP-CATCH trial starting in August 2018. Recruitment approaches included posting to pediatric QI listservs, emailing practices that participated in prior QICs with the research team, and direct referral. Practices attended a 1-hour webinar in December 2018 to review the purpose of the study and the medical record review tool. A total of 64 practices were randomized, but 59 submitted baseline data from clinical encounters documented between November 2018 and January 2019 (eFigure in Supplement 2). Participating practices had the opportunity to earn continuing medical education, maintenance of certification, and continuing education unit credit by attending webinars and engaging in QI activities, and practices were given an incentive to offset costs related to data entry. A listserv and research staff were available to answer questions about data entry. Outlier data were identified on a monthly basis and referred back to sites for confirmation.

Practices identified the first 17 eligible patients with BP measurements in the elevated or higher range each month based on medical record reviews. They then entered clinical encounter data to assess guideline adherence. Patients were excluded if they were presenting for a sick visit, were previously diagnosed with hypertension or an elevated BP, or had a prior diagnosis of congenital heart disease, chronic kidney disease, urologic disease, an organ transplant, or an extremely elevated BP or symptoms requiring emergency care. Patients with eating disorders were not specifically excluded.

### Measures

Baseline measures were extracted from medical records, including demographics (sex, age, race, ethnicity, and insurance types), anthropometric measures (weight in kilograms and height in meters), and systolic and diastolic BPs. All body mass index (BMI; calculated as weight in kilograms divided by height in meters squared) metrics—BMI, BMI *z* score, and BMI percentile—were computed by the research team based on medical record–extracted sex, age, weight, and height following the CDC 2000 growth chart calculation algorithm.^35^ Weight status classifications—underweight, normal, overweight, and obesity—were then made by the research team following the CDC criteria based on BMI percentiles.^33,36^ Specifically, overweight was defined as a BMI at or above the 85th percentile but below the 95th percentile and obesity as a BMI at or above the 95th percentile. Blood pressure classifications—elevated BP, stage 1 hypertension, and stage 2 hypertension—were also made by the research team based on medical record–extracted sex, age, and systolic or diastolic BP following the CDC guidelines^19,37^ for pediatric populations using the Rosner algorithm for computing age-, sex-, and height-specific BP percentiles.^38^

### Racial and Ethnic Groups

The race and ethnicity of participants were extracted from medical records by a study coordinator at each practice. We combined Hispanic ethnicity and race measures into the following 4 groups: Hispanic, non-Hispanic Black (hereafter, *Black*), non-Hispanic White (hereafter, *White*), and other, which included Asian, multiracial, other races, and unknown race.

### Counseling Topics

Practices were asked to indicate whether counseling regarding nutrition, lifestyle modifications, and/or weight was documented. The AAP recommends that patients with a high BP measurement receive (1) nutrition counseling, with a specific emphasis on the Dietary Approaches to Stop Hypertension (DASH) diet; (2) lifestyle counseling on physical activity and sleep; and (3) weight counseling if the patient has overweight or obesity.

Whether or not a patient received counseling on these 3 topics was tracked, with each topic as a separate measure and also all 3 topics in combination (ie, did a patient receive all counseling topics as appropriate? [yes or no]). If a patient did not have an overweight or obese weight status, practices were given credit for completing all counseling topics if only nutrition and lifestyle modification counseling were performed. The counseling could be delivered by any qualified practitioner, including but not limited to physicians, physician associates or assistants, nutritionists, nurse practitioners, and registered dietitians as long as it was documented in the medical record at that visit. In short, receiving counseling from a patient’s standpoint was equivalent to delivering guideline-concordant or compliant care from a practitioner’s standpoint. It is unknown, however, whether parings or dyads between patients and counseling practitioners were made based on race, sex, ethnicity, or other characteristics. In addition, although the BP-CATCH team provided general guidance on appropriate nutrition counseling (eg, DASH diet) in the context of high BP, there was no standardized or protocolized educational intervention.

### Statistical Analysis

This analysis was conducted from October 2023 to July 2024. Descriptive statistics included frequencies, percentages, means, and SDs. Baseline demographics, anthropometric measures, and BP measures were compared across the 4 racial and ethnic groups using χ^2^ or analysis of variance tests. Adjusted estimates and 95% CIs were obtained based on generalized mixed-effects models with clinic-level random intercepts that controlled clustering effects of clinics or practices for testing significance of difference in rates of receiving counseling across the 4 racial and ethnic groups. Post hoc pairwise comparisons were made to identify pairs of racial and ethnic groups that had significantly different rates of receiving counseling after Bonferroni adjustments to *P* values for the multiple testing were applied. All models included age, sex, insurance type, and BP classification as a set of covariates for adjustment. All of these analyses were further conducted with stratification by obese (BMI ≥95th percentile) and nonobese (BMI <95th percentile) weight status. Effect sizes were quantified in terms of risk differences (RDs), along with 95% CIs, in outcome rates between pairs of groups. Data were analyzed using SAS, version 9.4 (SAS Institute Inc). Statistical significance was declared when a 2-sided *P* value was less than .05.

## Results

### Baseline Characteristics

Although a total of 2855 medical records from visits were reviewed during the baseline period (eFigure in Supplement 2), only data from the first visits from 2677 patients were analyzed (Table 1). Mean (SD) patient age was 10.8 (5.2) years; 1161 (43.4%) were female; 1516 (56.6%), male; 593 (22.1%), Black; 414 (15.5%), Hispanic; 1111 (41.5%), White; and 559 (20.9%), other race and ethnicity. A total of 1362 patients (50.9%) were covered by private insurance, 785 of 2536 (31.0%) had obesity, and 1277 (47.7%) had stage 1 or 2 hypertension (Table 1). Except for sex and stage 1 hypertension, all baseline characteristics were significantly different across the 4 groups. Detailed comparisons of characteristics by racial and ethnic groups are presented in Table 1. For instance, the Hispanic group had the highest obesity rate (170 of 390 [43.6%]) and lowest stage 2 hypertension rate (9 of 414 [2.2%]), whereas the White group had the lowest obesity rate (260 of 1030 [25.2%]) and the Black group had the highest stage 2 hypertension rate (39 of 593 [6.6%]).

**Table 1.** Distributions of Baseline Characteristics by Race and Ethnicity

Characteristic	Participants^a^					*P* value^c^
	All (N = 100)	Black (n = 593)	Hispanic (n = 414)	White (n = 1111)	Other (n = 559)^b^	
**Demographic profiles**						
Age, mean (SD), y	10.8 (5.2)	11.0 (5.3)	10.0 (5.1)	11.5 (5.3)	9.9 (4.9)	<.001
Sex						
Female	1161/2677 (43.4)	261/593 (44.0)	165/414 (39.9)	486/1111 (43.7)	249/559 (44.5)	.47
Male	1516/2677 (56.6)	332/593 (56.0)	249/414 (60.1)	625/1111 (56.3)	310/559 (55.5)	
Private insurance	1362/2677 (50.9)	160/593 (27.0)	112/414 (27.1)	785/1111 (70.7)	305/559 (54.6)	<.001
**Anthropometric profiles**						
Weight, mean (SD), kg	49.5 (30.0)	52.9 (33.0)	47.6 (29.2)	50.6 (29.1)	45.0 (28.3)	<.001
Height, mean (SD), cm	140.8 (28.0)	142.1 (28.1)	136.1 (27.2)	143.6 (28.1)	137.3 (27.5)	<.001
BMI						
Mean (SD)	22.4 (8.4)	23.5 (10.0)	22.8 (8.7)	22.1 (8.1)	21.2 (6.5)	<.001
*z* Score, mean (SD)	0.9 (1.2)	1.0 (1.2)	1.2 (1.2)	0.7 (1.1)	0.8 (1.2)	<.001
Percentile, mean (SD)	71.9 (28.8)	73.5 (28.6)	78.8 (26.2)	68.9 (29.1)	70.7 (29.4)	<.001
Obesity^d^	785/2536 (31.0)	196/565 (34.7)	170/390 (43.6)	260/1030 (25.2)	159/551 (28.9)	<.001
**Blood pressure profiles**						
Blood pressure						
Systolic, mean (SD)	114.9 (12.4)	115.7 (12.7)	114.0 (11.3)	115.4 (13)	113.9 (11.5)	.02
Diastolic, mean (SD)	70.6 (8.2)	72.0 (8.5)	70.6 (7.7)	70.1 (8.2)	69.9 (7.9)	<.001
Elevated, mean (SD)	1400/2677 (52.3)	269/593 (45.4)	223/414 (53.9)	605/1111 (54.5)	303/559 (54.2)	.002
Hypertension stage						
1	1161/2677 (43.4)	285/593 (48.1)	182/414 (44.0)	460/1111 (41.4)	234/559 (41.9)	.05
2	116/2677 (4.3)	39/593 (6.6)	9/414 (2.2)	46/1111 (4.1)	22/559 (3.9)	.007

Abbreviation: BMI, body mass index (calculated as weight in kilograms divided by height in meters squared).

^a^ Data are presented as number/total number (percentage) of participants unless otherwise indicated.

^b^ Includes Asian, multiracial, other races, and unknown race.

^c^ For testing equality of outcomes across the 4 racial and ethnic groups based on analysis of variance and χ^2^ tests.

^d^ BMI percentile or 95 or higher.

### Nutrition Counseling

**Figure 1.**
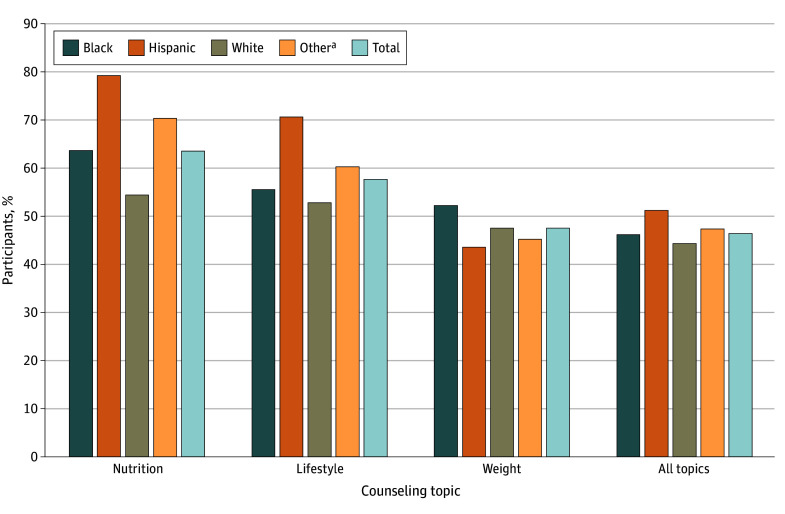
Crude Unadjusted Rates of Receiving Counseling Across the 4 Race and Ethnicity Groups and Among All Participants ^a^Other includes Asian, multiracial, other races, and unknown race.

**Figure 2.**
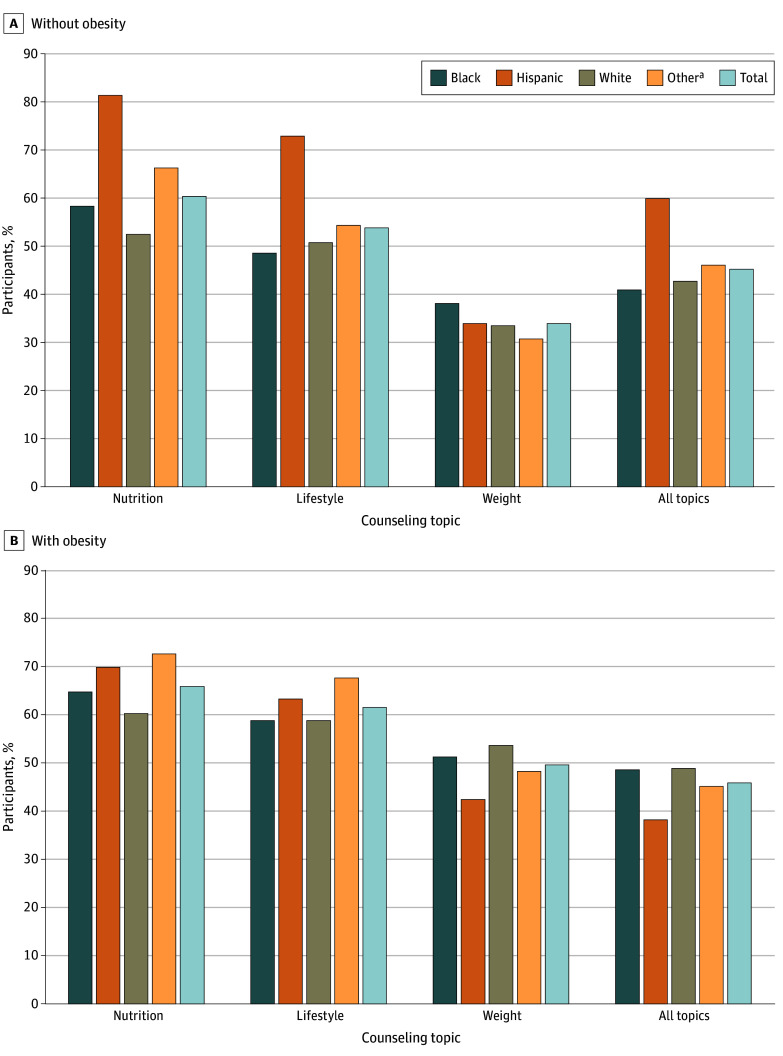
Crude Unadjusted Rates of Receiving Counseling Across the 4 Race and Ethnicity Groups and All Participants by Weight Status ^a^Other includes Asian, multiracial, other races, and unknown race.

The crude unadjusted rates of receiving nutrition counseling (overall, 1564 of 2463 [63.5%]) among all participants are presented in Figure 1, with the highest rate among Hispanic participants (294 of 371 [79.2%]). The adjusted estimated rates were significantly different across the 4 racial and ethnic groups (eg, Hispanic participants, 78.6% [95% CI, 73.5%-83.8%]; White participants, 54.6% [95% CI, 50.4%-58.8%]; *P* < .001) (Table 2). The Hispanic group had significantly higher rates of receiving nutrition counseling compared with all other race and ethnicity groups (eg, adjusted RD [ARD] compared with White participants, 24.0% [95% CI, 18.6%-29.5%]; *P* < .001) (Table 3). The White group had significantly lower rates of nutrition counseling compared with all other race and ethnicity groups (eg, compared with Black participants: ARD, 8.6% [95% CI, 3.3%-14.0%]; *P* = .01) (Table 3). This overall pattern was consistent among participants without obesity (Figure 2A) except that there was no significant difference between the Black and White groups(Table 3). The rate of receiving nutrition counseling among participants with obesity was highest in the other race and ethnicity group (Figure 2B). Although the adjusted rates of receiving nutrition counseling among participants with obesity were significantly different across the 4 racial and ethnic groups (Table 2), a significant difference was observed only between White participants (68.3% [95% CI, 61.1%-75.4%]) and those reporting other race and ethnicity (81.6% [95% CI, 74.2%-89.1%]) (ARD, −13.4% [95% CI, −22.2% to −4.6%]; Bonferroni-corrected *P* = .02) (Table 2 and Table 3).

**Table 2.** Estimated Adjusted Rates of Receiving Counseling Across 4 Racial and Ethnic Groups

Counseling topic	Adjusted rate, % (95% CI)				*P* value^b^
	Black	Hispanic	White	Other^a^	
**All participants**					
Nutrition	63.2 (58.4-68.1)	78.6 (73.5-83.8)	54.6 (50.4-58.8)	69.6 (64.7-74.5)	<.001
Lifestyle	54.4 (49.4-59.5)	69.3 (63.6-74.9)	51.1 (46.8-55.4)	58.7 (53.5-63.9)	<.001
Weight	54.4 (47.7-61.2)	48.7 (40.8-56.6)	49.6 (43.8-55.4)	49.5 (42.4-56.6)	.53
All topics	47.0 (41.9-52.0)	52.1 (46.1-58.2)	43.7 (39.4-48.0)	47.6 (42.3-52.8)	.06
**Participants without obesity**					
Nutrition	55.9 (49.5-62.4)	77.7 (70.7-84.7)	49.4 (43.8-54.9)	62.6 (56.1-69.0)	<.001
Lifestyle	46.2 (39.7-52.6)	68.9 (61.3-76.4)	45.0 (39.5-50.4)	49.4 (42.9-55.9)	<.001
Weight	34.8 (22.8-46.8)	31.2 (16.1-46.3)	26.7 (17.1-36.3)	27.4 (15.9-38.9)	.66
All topics	39.4 (33.1-45.7)	57.4 (49.4-65.4)	37.2 (31.9-42.6)	41.8 (35.4-48.3)	<.001
**Participants with obesity**					
Nutrition	73.4 (65.7-81.2)	79.0 (70.9-87.1)	68.3 (61.1-75.4)	81.6 (74.2-89.1)	.02
Lifestyle	66.3 (58.0-74.6)	71.3 (62.4-80.2)	66.8 (59.4-74.2)	75.8 (67.7-83.9)	.20
Weight	61.5 (53.1-69.8)	54.3 (44.9-63.7)	63.5 (56.2-70.8)	58.8 (49.9-67.7)	.37
All topics	56.8 (48.2-65.4)	47.1 (37.6-56.6)	57.6 (49.9-65.2)	53.7 (44.6-62.8)	.22

^a^ Includes Asian, multiracial, other races, and unknown race.

^b^ For testing equality of outcome rates across the 4 racial and ethnic groups based on generalized mixed-effects models adjusting for age, sex, insurance type, and blood pressure classification.

**Table 3.** Estimated Pairwise ARDs in Rates of Receiving Counseling Across 4 Race and Ethnicity Groups

Race and ethnicity group^a^	All participants		Nonobese weight status		Obese weight status^b^	
	ARD (95% CI), %	*P* value^c^	ARD (95% CI), %	*P* value^c^	ARD (95% CI), %	*P* value^c^
**Nutritional counseling**						
Black compared with Hispanic	−15.4 (−21.2 to −9.6)	<.001	−21.7 (−29.3 to −14.2)	<.001	−5.6 (−3.8 to 15.0)	>.99
Black compared with White	8.6 (3.3 to 14.0)	.01	6.6 (−0.3 to 13.4)	.36	5.1 (−3.9 to 14.2)	>.99
Black compared with other	−6.4 (−12.2 to −0.5)	.19	−6.6 (−14.0 to 0.8)	.48	−8.2 (−17.5 to 1.1)	.49
Hispanic compared with White	24.0 (18.6 to 29.5)	<.001	28.3 (21.4 to 35.2)	<.001	10.7 (1.4 to 20.1)	.14
Hispanic compared with other	9.1 (3.2 to 14.9)	.01	15.1 (7.7 to 22.5)	<.001	−2.6 (−12.0 to 6.8)	>.99
White compared with other	−15.0 (−20.0 to −9.9)	<.001	−13.2 (−19.3 to −7.0)	<.001	−13.4 (−22.2 to −4.6)	.02
**Lifestyle counseling**						
Black compared with Hispanic	−14.8 (−21.1 to −8.6)	<.001	−22.7 (−31.0 to −14.5)	<.001	−5.0 (−15.2 to 5.2)	>.99
Black compared with White	3.3 (−2.2 to 8.8)	>.99	1.2 (−5.7 to 8.1)	>.99	−0.5 (−9.9 to 9.0)	>.99
Black compared with other	−4.3 (−10.4 to 1.8)	>.99	−3.2 (−10.9 to 4.4)	>.99	−9.5 (−19.5 to 0.6)	.38
Hispanic compared with White	18.1 (12.3 to 24.0)	<.001	23.9 (16.4 to 31.4)	<.001	4.6 (−5.4 to 14.5)	>.99
Hispanic compared with other	10.6 (4.1 to 17.0)	.008	19.5 (11.3 to 27.7)	<.001	−4.5 (−14.8 to 5.9)	>.99
White compared with other	−7.6 (−12.9 to −2.3)	.03	−4.4 (−10.8 to 1.9)	>.99	−9.0 (−18.4 to 0.3)	.35
**Weight counseling**						
Black compared with Hispanic	5.7 (−3.1 to 14.6)	>.99	3.6 (−12.8 to 20.0)	>.99	7.1 (−3.7 to 18.0)	>.99
Black compared with White	4.8 (−2.8 to 12.5)	>.99	8.1 (−5.0 to 21.2)	>.99	−2.1 (−11.8 ot 7.6)	>.99
Black compared with other	4.9 (−3.8 to 13.6)	>.99	7.4 (−7.1 to 21.9)	>.99	2.7 (−8.3 to 13.6)	>.99
Hispanic compared with White	−0.9 (−9.4 to 7.6)	>.99	4.5 (−11.2 to 20.2)	>.99	−9.2 (−19.8 to 1.4)	.54
Hispanic compared with other	−0.8 (−10.1 to 8.5)	>.99	3.8 (−12.9 to 20.6)	>.99	−4.5 (−16.1 to 7.1)	>.99
White compared with other	0.1 (−7.7 to 7.9)	>.99	−0.7 (−12.8 to 11.4)	>.99	4.7 (−5.5 to 15.0)	>.99
**All counseling topics**						
Black compared with Hispanic	−5.2 (−11.8 to 1.5)	.76	−18.0 (−26.6 to −9.4)	<.001	9.7 (−1.2 to 20.5)	.48
Black compared with White	−3.3 (−8.8 to 2.2)	>.99	2.2 (−4.6 to 9.0)	>.99	−0.8 (−10.6 to 9.0)	>.99
Black compared with other	−0.6 (−6.8 to 5.6)	>.99	−2.4 (−10.0 to 5.1)	>.99	3.1 (−8.0 to 14.1)	>.99
Hispanic compared with White	8.4 (2.2 to 14.6)	.047	20.2 (12.2 to 28.2)	<.001	−10.4 (−21.0 to 0.1)	.32
Hispanic compared with other	4.6 (−2.2 to 11.4)	>.99	15.6 (6.9 to 24.2)	.003	−6.6 (−18.1 to 5.0)	>.99
White compared with other	−3.9 (−9.2 to 1.5)	.95	−4.6 (−11.0 to 1.7)	.92	3.8 (−6.5 to 14.2)	>.99

Abbreviation: ARD, adjusted risk difference.

^a^ Other race and ethnicity includes Asian, multiracial, other races, and unknown race.

^b^ Obese weight status was classified as body mass index (calculated as weight in kilograms divided by height in meters squared) in the 95th percentile or greater. The 95% CIs are not Bonferroni-adjusted.

^c^ All *P* values are Bonferroni adjusted for 6 post hoc pairwise tests for each counseling outcome based on generalized mixed-effects models adjusting for age, sex, insurance type, and blood pressure classification.

### Lifestyle Counseling

The crude unadjusted rates (overall, 1419 of 2462 [57.6%]) of receiving lifestyle counseling among all patients are presented in Figure 1, again with the highest rate in the Hispanic group (262 of 371 [70.6%]). The adjusted estimated rates were significantly different across the 4 racial and ethnic groups (Table 2). The Hispanic group had significantly higher adjusted rates of lifestyle counseling (69.3% [95% CI, 63.6%-74.9%]) compared with all other racial and ethnic groups, and the White group had significantly lower adjusted rates of lifestyle counseling (51.1% [95% CI, 46.8%-55.4%]) (*P* < .001 for all comparisons). A significant difference was found between Hispanic participants and White participants (ARD, 18.1% [95% CI, 12.3%-24.0%]; Bonferroni-corrected *P* < .001) and between White participants and those reporting other race and ethnicity (adjusted rate, 58.7% [95% CI, 53.5%-63.9%]; ARD, −7.6% [95% CI, −12.9% to −2.3%]; Bonferroni-corrected *P* = .03) (Table 3).

Hispanic participants had the highest rate of lifestyle counseling (146 of 200 [73.0%]) among those without obesity (Figure 2A), but the other race and ethnicity group had the highest rate among those with obesity (108 of 142 [76.1%]) (Figure 2B). Among participants without obesity, the Hispanic group had a significantly higher adjusted rate compared with all other groups (Table 2), but there were no significant differences between any pairs of the other 3 groups excluding the Hispanic group (Table 3). However, among participants with obesity, the adjusted rates were not significantly different across the 4 groups (Table 2) or among any pair of groups (Table 3).

### Weight Counseling

The crude unadjusted rates of receiving weight counseling (overall, 571 of 1202 [47.5%]) among all participants are presented in Figure 1, with the highest rate in the Black group (153 of 293 [52.2%]), which also had the highest rate of weight counseling among those who had overweight but not obesity (Figure 2A), whereas the White group had the highest rate of weight counseling (146 of 242 [60.3%]) among participants with obesity (Figure 2B). When stratified by weight status, the overall unadjusted rate was 55.8% (400 of 717) among participants with obesity and 34.0% (136 of 400) among those with overweight. Overall and when accounting for patients’ weight status, however, the adjusted rates were not significantly different across the 4 groups (Table 2) or among any pair of groups (Table 3).

### All Counseling Topics

The crude unadjusted rates of receiving all counseling topics (overall, 1142 of 2461 [46.4%] received 2 or 3 of the counseling interventions depending on weight status) among all participants are presented in Figure 1, with the highest rate in the Hispanic group (190 of 371 [51.2%]). Although the adjusted rates were not significantly different across the 4 groups (Table 2), a significant difference was observed between Hispanic and White groups (43.7% [39.4%-48.0%] and 52.1% [95% CI, 46.1%-58.2%], respectively; ARD, 8.4% [95% CI, 2.2%-14.6%]; Bonferroni-corrected *P* = .047) (Table 3).

The crude unadjusted rate among participants without obesity was highest in the Hispanic group (120 of 200 [60.0%]) (Figure 2A), whereas among participants with obesity, it was highest in the White group (133 of 242 [55.0%]). The adjusted rates were significantly different across the 4 groups among participants without obesity but not among participants with obesity (Table 2). Specifically, among participants without obesity, the Hispanic group had a significantly higher adjusted rate (57.4% [95% CI, 49.4%-65.4%]) compared with the Black group (39.4% [95% CI, 33.1%-45.7%]; *P* < .001; ARD, −18.0% [95% CI, −26.6% to −9.4%]; Bonferroni-corrected *P* < .001) and the White group (37.2% [95% CI, 31.9%-42.6%]; *P* < .001; ARD, 20.2% [95% CI, 12.2%-28.2%]; Bonferroni-corrected *P* < .001) (Table 3). No significant differences in adjusted rates were observed among any pair of groups among participants with obesity (Table 3).

## Discussion

The primary finding from the present study is that the overall rate of receiving counseling among children with high BP appeared to be suboptimal and varied not only by counseling topics but also by race and ethnicity and weight status. The overall rates for receiving nutrition and lifestyle counseling were 63.5% and 57.6%, respectively, whereas for receipt of weight counseling and all counseling topics, the rates were 47.5% and 46.4%, respectively. This pattern was consistent among participants without obesity, but almost all rates were slightly above 50% among participants with obesity. Of note, only 55.8% of participants with obesity and 34.0% who were overweight received weight counseling during primary care visits.

The Hispanic group had the highest rates of nutrition and lifestyle counseling compared with all other racial and ethnic groups. Nevertheless, there were no significant racial and ethnic disparities for weight counseling or all counseling topics except that the Hispanic group had a higher rate of receiving all counseling topics compared with the White group. This finding was unexpected given that the Hispanic group did not have the highest BP measurements or hypertension rates, although this group had the highest obesity rate; that is, the Hispanic group received significantly more nutrition and lifestyle counseling but not weight counseling. It is possible that Hispanic patients were more likely to use English as a second language, potentially complicating counseling for some practitioners speaking English only. This notion may highlight the importance of concordant pairing between Hispanic patients and practitioners based on primary languages or the availability or presence of a trained or certified interpreter during visits in practices. Further refined counseling modalities could be developed that are tailored to sex, birth place (US or non-US born), acculturation levels, and eating practices.^39,40^ Among participants with obesity, however, the rate of receiving all counseling topics was less than 50% only for Hispanic participants, who also had the lowest rate of receiving weight counseling despite this group having the highest obesity rate. In contrast, rates of receiving weight counseling and all topics of counseling among participants with obesity were highest for the White group, although the obesity rate in this group was lowest.

Although potential underlying reasons for the aforementioned racial and ethnic disparities are unknown, the findings are somewhat consistent with an earlier study conducted by Kallem et al^41^ that reported racial disparities in counseling rates and BMI status. In that study, Hispanic and Black participants were more likely to receive counseling than White participants. Nonetheless, the study reported that 23.9% of children with obesity did not receive weight counseling,^41^ whereas 39.7% to 52.7% of participants with obesity in our study did not receive weight counseling across racial and ethnic groups. A possible reason for the higher rate of nonadherence observed in our study could be that our outcomes were based on medical record reviews from clinical practices, whereas the study by Kallem et al^41^ was conducted among school children and relied on self-report from the children, potentially leading to social desirability and recall bias.

With approximately half of this study’s sample population overall receiving each of the recommended counseling topics, it appears to be necessary to identify obstacles in adhering to the AAP guidelines. There is a possibility that barriers to receiving counseling may originate from the patient or family, and these barriers could include cultural beliefs, social determinants of health, and language differences, among others. Health care practitioners may also benefit from culturally sensitive education and trainings to find ways to balance patients’ health needs and cultural beliefs about eating attitudes, lifestyle, and body satisfaction.^42^ Further research seems to be warranted to identify barriers to counseling pediatric patients from the standpoint of health care professionals, the health system, patients themselves, and their families.

### Strengths and Limitations

Our study has the following strengths. A large number of pediatric practices across the US with a variety of practice settings and sizes were enrolled. To maximize the quality of data collected, the BP-CATCH research team conducted regular monthly data collection and training webinars, answered questions on listservs, requested clarification for grossly abnormal data entry, and shared clarifications regarding data collection processes in a uniform manner across practices as much as possible. Furthermore, the BP-CATCH study involved both primary care practices and local subspecialists with expertise in pediatric hypertension, which reflects the collaborative nature of care for this population.

The following limitations should be taken into account when interpreting our study findings. As practices voluntarily self-enrolled in the BP-CATCH QIC, participating practices may not be a representative sample of all pediatric primary care settings in the US. Moreover, each practice collected their own data based on medical record reviews in a convenient fashion, rather than data being randomly extracted by an independent research team. Neither patients’ nor practitioners’ potential refusal or inability to receive or deliver counseling was noted. We also did not have information on participants’ preferred languages. Finally, although the BP-CATCH trial included practices from across the US, the distribution of race and ethnicity in our data are slightly different from the US as a whole.

## Conclusions

This secondary analysis of the BP-CATCH randomized clinical trial baseline data found that in a pediatric population with elevated or higher BP, the overall rates of receiving counseling recommended to mitigate hypertension and obesity appeared to be suboptimal. Racial and ethnic disparities in receiving nutrition, lifestyle, and all counseling topics were significant, and the patterns of disparities were inconsistent between participants with and without obesity. More effort may need to be invested into developing QI or other interventions to promote all topics of recommended counseling, perhaps more intensively among children with obesity, and to attenuate racial and ethnic disparities in pediatric primary care settings. Our study also suggests that quality metrics should be stratified by demographic categories to highlight disparities and prevent worsening of inequities as efforts continue to improve and standardize care for all children.

## Notes

Supplement 1.
Trial Protocol

Supplement 2. **eFigure.** CONSORT diagram

Supplement 3.
Data Sharing Statement
