# Supplementary material for: Racial and Ethnic Disparities in Pediatric Counseling on Nutrition, Lifestyle, and Weight: A Secondary Analysis of the BP-CATCH Randomized Clinical Trial
Source: JAMA Netw Open. 2025 Jan 29;8(1):e2456238. doi: 10.1001/jamanetworkopen.2024.56238 (PMC11780477; doi:10.1001/jamanetworkopen.2024.56238)
Supplement: Supplement 1. — Trial Protocol [file jamanetwopen-e2456238-s001.pdf]

BP-CATCH:
Boosting Primary Care Awareness and Treatment of Childhood Hypertension

Sponsor

Children's Hospital at Montefiore

3411 Wayne Avenue, NY, United States

Protocol Version

February 16, 2021

version 10

Confidentiality Statement:

This document contains confidential information that must not be disclosed to anyone other than the Sponsor, the Investigator Team, HRA, host organisation, and members of the Research Ethics Committee, unless authorised to do so.

Research Team:

Principal Investigator: Michael L. Rinke, MD, PhD,

Co-investigators at Einstein/Montefiore: Beatrice Goilav, MD

Collaborating Centers investigators:

- David G. Bundy, MD, MPH, Medical University of South Carolina (MUSC)
- Tammy M. Brady, MD, PhD, Johns Hopkins University School of Medicine
- Beth Tarini, MD, Children’s National Medical Center
- Katherine E. Twombley, MD, Medical University of South Carolina (MUSC)
- Corrina Rea, MD MPH Boston Children’s Hospital
- Kelly Orringer, MD, University of Michigan
- Kimberly Giuliano, MD Cleveland Clinic
- Moonseong Heo, PhD / Biostatistician, Clemson University

Research support staff: Peterkaye Kelly, MPH

Table of contents

1- Significance and Background 4
2- Study aims 7
3- Study Design, Cohort, and Methodology for Quality Improvement Collaborative 8
4- Pediatric HTN Outcome Measures 13
5- Data and Safety Monitoring Plan 15
6- Statistical Analysis 17
7- Risks and Benefits 19
8- Informed Consent 21
9- Confidentiality 21
10-Appendix 27

1. SIGNIFICANCE AND BACKGROUND:

The proposed research, building on an ongoing AHRQ-funded research project to prevent pediatric diagnostic errors in primary care (R01HS023608) and using a prospective, cluster-randomized, stepped wedge design, will investigate whether 1) a quality improvement collaborative (QIC) intervention without subspecialist involvement, 2) a QIC with subspecialists and primary care physicians (PCPs) mutually engaged, and/or 3) a hub and spoke co-diagnosis, co-management model where PCPs diagnose and manage pediatric hypertension (HTN) with a supporting subspecialist advisor, reduce errors in pediatric HTN diagnosis and management compared to each other and usual care. We will also investigate time to HTN diagnosis and management, PCP and subspecialist satisfaction, and correct blood pressure (BP) measurement procedures. This project will be carried out in a national group of primary care practices in urban, rural and suburban locations. This approach, integrating PCP and subspecialty care physicians, could serve as a model across the spectrum of pediatric chronic diseases.

1.1. Summary of Significance

Pediatric HTN causes appreciable morbidity in pediatric patients and errors in diagnosis and management are frequent and understudied, jeopardizing pediatric safety in ambulatory settings. Additionally, the gap between the number of pediatric subspecialist providers and the number needed for patient care continues to widen, and it is unclear how to best reduce burden on subspecialists, improve PCP and subspecialist communication, and improve patient outcomes. This research team, with significant experience researching ambulatory pediatric safety, conducting QICs and HTN interventions, identified six large pediatric practice groups in rural, suburban and urban locations that are committed to reducing preventable HTN patient harm, to testing the effectiveness of a QIC to improve PCP HTN diagnosis and management, and to a hub and spoke HTN co-diagnosis and co-management model. The effect demonstrated by this project using a rigorous research design and the new 2017 pediatric HTN guidelines,[6] will motivate pediatric clinics across the country to adopt these newly-identified best practices to improve pediatric HTN care. Primary care pediatricians have an imperative to diagnose and manage HTN and elevated BP (EBP) more accurately and earlier, and to improve interactions with subspecialists to reduce the lifelong preventable harm that results from these chronic conditions. This proposal, will identify a clear implementation strategy for rigorous, evidenced-based pediatric HTN diagnosis and management, and highlight a model to increase primary and subspecialty care integration that can be reproduced across other chronic conditions.

1.2. Study Overview and Rationale:

This proposal aims to test whether a QIC without and with subspecialist involvement, and/or a hub and spoke PCP-subspecialty care co-diagnosis and co-management model can improve pediatric HTN care. We propose to conduct a prospective, cluster-randomized stepped wedge trial, broadening and deepening our prior work on recognition of pediatric elevated BP, which will test methodologies to ensure every pediatric patient is screened, and if EBP or HTN are present, diagnosed and managed appropriately and expeditiously. These data will inform future efforts to improve care delivery and safety for all pediatric chronic conditions, provide broad generalizability on AHRQ priority populations including rural and urban children, and generate broader data on QIC efforts aimed at pediatric HTN. Control group data from the QIC will identify incidence rates for all phases of pediatric HTN diagnosis and management errors. The proposal will also serve as a test case for whether pediatricians can manage common conditions (e.g. HTN) with a supporting

subspecialist advisor. Human subjects' protection review will be completed at Montefiore/Einstein and at each individual site as needed with assistance from the PI and the program manager.

The first step in making a HTN or EBP diagnosis is recognizing when pediatric BP is elevated. While more straightforward in adults, BP measurement in children is challenging as it requires specialized skills including 1) preparing and calming a child; 2) choosing an appropriately sized cuff, and 3) interpreting the BP based on burdensome age, sex and height tables.[7] A study by a member of this project's leadership team demonstrated that 39% of children have elevated BP readings at pediatric visits but 87% of these elevations are not recognized by the PCP.[8] Other studies found that 74% of children with HTN or EBP were misdiagnosed by pediatricians,[9] and that 47% of pediatricians classified one or more elevated BP readings as normal.[10] Misdiagnosed pediatric HTN has appreciable cardiovascular consequences for children including increased rates of progression towards insulin resistance, atherosclerosis and metabolic syndrome. Pediatric HTN is also associated with increased risk of adult HTN.[11-14] Additionally, studies demonstrate EBP and HTN cause irreversible cardiovascular damage in children[15,16] suggesting diagnosis and management occur too late in the disease process. Guidelines for pediatric HTN and EBP were recently updated.[6] To our knowledge this is the first proposal which will implement the new HTN guidelines[6] and improve pediatric HTN diagnosis and management.

The number of pediatric ambulatory care visits resulting in a subspecialist referral more than doubled in 10 years to 10.5 million annually in 2009,[18] suggesting an increasing need for pediatric PCP and subspecialty care integration. Unfortunately, pediatric subspecialists are often maldistributed and frequently have long waits for visits, even in urban areas with higher subspecialist concentration.[19-22] Additionally, the increasing sub-specialization of pediatric care due to increasing technology and medico-legal concerns may unintentionally reduce the scope of pediatric PCP practice, and overburden subspecialists with patients not suited for their clinics.[21,23] When children do access care, communication is often poor: PCPs report not being able to access subspecialist plans of care, parents are expected to communicate between medical disciplines, and children are left with incompletely addressed medical sequelae.[24-27] Almost two-thirds of PCPs report being dissatisfied with the referral process, more than two-thirds of subspecialists report receiving no information from the PCP, and one-quarter of PCPs receive no subspecialist feedback after four weeks.[28] Families similarly identify multiple obstacles to excellent subspecialty care.[29] As the mismatch between pediatric subspecialist need and pediatric subspecialist availability grows, children with chronic conditions will be increasingly subjected to suboptimal medical care and health outcomes.[30]

While adult patients with HTN are commonly managed by PCPs, pediatric HTN patients are commonly referred to pediatric nephrologists and/or cardiologists,[31] despite the fact that 66%-80% of pediatric patients can likely be managed using evidenced-based algorithms in a primary care setting.[32-34] The majority of pediatric HTN subspecialists agree that PCPs can make the diagnosis of HTN independently, and cite over-referral of patients due to inaccurate BP readings as a problem in their clinics.[35] Given the harmful sequelae, it is essential that we improve PCP and subspecialist care integration for pediatric chronic conditions like HTN.

One possible solution to the pressing need for pediatric subspecialist care and improved PCP and subspecialist care integration is a 'hub and spoke' co-diagnosis and co-management model, where one subspecialist advises and consults with multiple PCPs. In the hub and spoke model, the patient is seen by a PCP in the primary care setting, reducing wait times and transportation costs, especially for rural patients. The PCP is supported by a subspecialist who can help develop algorithms for care before patient encounters, aid the PCP with unexpected findings and questions as needed. The hub and spoke model was successfully utilized for treatment of patients with *human immunodeficiency virus* (HIV) at the PI's current institution.[36,37] Researchers found no difference in HIV patient outcomes comparing those treated at a subspecialist HIV clinic and those treated by a PCP with HIV subspecialist support.[36,37] The hub and spoke model has also been successfully

employed to teach pediatric quality improvement.[38] A co-investigator on this grant, Dr. Elissa Faro, employed the hub and spoke model to improve PCP management of patients with sickle cell disease, another pediatric chronic condition. Other examples of pediatric hub and spoke care exist and suggest benefit, although small sample sizes, short follow-up times, and lack of rigorous statistical testing reduces the generalizability of these preliminary works.[30] To clarify, a hub and spoke model differs from telemedicine[39,40] because its goal is for the PCP to become increasingly comfortable with diagnosing and managing patients with a given chronic condition, not just improving access to subspecialists who still require the time and availability to meet with a patient via a telemedicine technology platform. Many PCPs support increasing autonomy with chronic care conditions,[21] including HTN,[41] and the hub and spoke model has the potential to 1) free subspecialists to see more complex patients, 2) allow PCPs to deliver guideline-based and equally efficacious care, and 3) improve patient safety by providing expeditious chronic care management.

One of the central challenges to diagnosing pediatric patients with hypertension is the need for three sequential office visits with elevated BP measurements documented to confirm that the BP elevations are sustained. Many patients do not return for second or third BP measurements because they are asymptomatic. In the first year of the BP-CATCH study, we observed this problem first hand as over 75% of patients did not return on time for their second or third BP measurement. This problem is exacerbated by the COVID-19 pandemic and the resulting telemedicine environment, as it becomes even harder for patients to feel safe to return to a clinical site solely for BP measurements.

One potential solution to this problem is confirm the blood pressure elevations in a home environment with home blood pressure monitoring (HBPM). This approach minimizes the need for patients to return for an in-person repeat and confirmatory BP measurement and is family-centered by acknowledging hesitancy to return to clinical environments during COVID-19. HBPM uses an automated BP device to measure resting out of office blood pressure in a standard way. Patients obtain three BP measurements each morning and three each evening for seven days. These measurements are then averaged to help determine if the patient has consistently elevated BPs which would warrant further evaluation and treatment. HBPM is not endorsed as a tool for the diagnosis of hypertension in the current evidenced based guidelines for pediatric hypertension due to insufficient evidence, but it is recommended as an adjunct for out of office management of children with an established diagnosis. Notably HBPM is included in the European pediatric hypertension guidelines as an adjunct for diagnosis, and is a guideline-based tool often used by adult practitioners to aid in the diagnosis and management of adult hypertension.

HBPM offers patients and providers an opportunity to continue to provide preventive care in an innovative, family-centered way during telemedicine visits or during any situation which makes in-person clinic visits challenging. Children and adolescents with elevated blood pressure could perform out-of-office blood pressure measurements via HBPM to determine if their BP elevations are sustained and warrant further evaluation. This strategy could bridge the gap of missed primary care prevention and could facilitate the diagnosis of this condition that affects 3-4 out of every 100 children in the US. Before broad implementation of this strategy, we need to understand how HBPM correlates with in-office manual BP measurements.

1.3 Preliminary Data and Effect Estimates:

Sigificant preliminary data has been collected to demonstrate the feasibility of identifying and reducing errors in HTN diagnosis and management through QIC methodologies. All tools and interventions utilized in the preliminary studies described will be available to the research team and participating practices, increasing the likelihood of success for this proposed research.

In 44 patients at Johns Hopkins with HTN or EBP, the mean time from first elevated BP reading until HTN diagnosis was 13 months. In the 23 HTN patients, the mean time was 16 months. In 35 patients at CHAM with HTN, the mean time was 46 days, and the mean time until initiation of

management was 68 days. This wide variation suggests a crucial need to standardize these practices across PCP and subspecialty care. Dr. Brady identified elevated BPs in 39% of pediatric ambulatory care visits but this abnormality was not recognized by providers in 87% of visits. Using an EHR intervention, Dr. Brady reduced this recognition error rate by 33%.[85]

Because our prior work only examined errors in elevated BP recognition, we conservatively estimated the baseline error rate for all steps in pediatric HTN diagnosis and management in naïve primary care practices to be higher at 73%. Given that our prior QIC reduced error rates by 48%, we hypothesize that multidisciplinary QIC interventions including evidenced-based tools and group learning, can at least reduce errors in pediatric HTN diagnosis and management similarly by 50%. Given the multidisciplinary nature of HTN diagnosis and management, we conservatively hypothesize that an additional 15% reduction in HTN error rates will occur with subspecialist integration into a QIC as compared to the original QIC without subspecialist involvement. Finally, we hypothesize a further 15% reduction will be realized by a hub and spoke co-diagnosis, co-management model as compared to QICs with HTN subspecialist involvement without focus on hub and spoke co-diagnosis and co-management.

2. Study Aims:

We propose to conduct a multisite, prospective, cluster-randomized trial, broadening and deepening our prior work on recognition of pediatric elevated BP, which will test methodologies to ensure every pediatric patient is screened, and if HTN or EBP are present, diagnosed and managed appropriately and expeditiously. This design, with practices randomized in a stepped-wedge fashion to a HTN QIC a) without subspecialist involvement, b) with subspecialist involvement and c) with pediatrician HTN diagnosis and management and subspecialist back-up, will also serve as a test case for whether pediatricians can diagnose and manage common chronic conditions (e.g., HTN) with a supporting subspecialist advisor. This proposal responds to AHRQ's Funding Opportunity Announcement PA-15-339, designed to develop evidence to improve safety in ambulatory care settings. This work is one of the first to test implementation of the new 2017 pediatric HTN guidelines and focuses on children in rural and urban settings, AHRQ priority populations.

Specific Aim 1: Determine whether a QIC in a national group of at least 60 pediatric practices, building on our prior elevated BP recognition work, is associated with reduction of errors in the broader outcomes of 1) HTN diagnosis, 2) initiation of management, and 3) time to diagnosis and management.

> <u>Hypothesis 1</u>: *Practices randomized to a QIC will experience a 50% reduction in pediatric HTN and EBP diagnosis and management errors as compared to usual care.*

Specific Aim 2: Investigate whether a QIC with relevant, local pediatric HTN subspecialist involvement improves diagnosis and management for children with HTN.

> <u>Hypothesis 2</u>: *Practices randomized to a collaborative with relevant, local HTN subspecialist involvement following participation in the original QIC, will experience an additional 15% reduction in pediatric HTN and EBP diagnosis and management errors as compared to the original QIC.*

Specific Aim 3: Determine whether pediatrician co-diagnosis and co-management of HTN, conducted via a 'hub and spoke' model where one pediatric HTN subspecialist advises multiple primary care pediatricians, improves diagnosis and management for children with HTN.

Hypothesis 3: Practices randomized to a HTN QIC with a hub and spoke model, following participation in the two QICs described above, will experience an additional 15% reduction in pediatric HTN and EBP diagnosis and management errors as compared to QICs with HTN subspecialist involvement but no focus on hub and spoke co-diagnosis and co-management.

Given the impact of COVID-19 on pediatric care across the country, the number of in-person pediatric visits has declined sharply. Many sites are now overwhelmed by changes to practice patterns due to COVID-19, and are being asked to see patients virtually. In this environment it is no longer feasible to run a QIC that relies on in-person pediatric BP measurements. For this reason, we paused the study on March 24, 2020. In discussions with our funder, AHRQ, and to continue this quality improvement work, we have created a modified aim as below:

Specific Aim 4: Determine whether it is feasible for families of children with one elevated blood pressure measurement to 1) accept home blood pressure monitoring (HBPM), 2) complete HBPM measurements, 3) return HBPM measurement data to clinicians, and 4) the correlation of these data with in-office manual BP measurements.

3. Study Design, Cohort, and Methodology for Quality Improvement Collaborative (QIC):

The goal of this proposal is to reduce the proportion of patients who experience pediatric HTN diagnosis and management errors, in order to reduce time to definitive management and therefore harmful sequelae often seen in children at the time of HTN diagnosis.[15,16] We will accomplish this goal and test whether a QIC which assists teams in reliably performing evidence-based HTN processes, a QIC with integrated PCP and subspecialist participation, and/or a hub and spoke PCP-subspecialist care model can improve these measures via a prospective, cluster-randomized, stepped wedge design. In the primary outcome analyses, each of these 3 conditions will be compared to each other and to usual care. Diagnosing and managing pediatric HTN involves multiple steps, multiple visits, and multiple decisions, and the 2017 AAP guidelines have recently updated these practices.[6] For this reason, and as demonstrated by the superior reduction in recognition of elevated BP error rates in this team's QIC as compared to Dr. Brady's EHR intervention, an EHR intervention or best practice alert alone would be insufficient to drive this level of behavior change; a QIC is needed.

Our cohort will consist of at least 60 pediatric primary care practice sites from at least 6, diverse practice groups: Cleveland Clinic Children's, Dartmouth Medical Center, Johns Hopkins Harriet Lane Clinic, Medical University of South Carolina (MUSC) and South Carolina Pediatric Research Network (SCPPRN), Montefiore Medical Group (MMG) and University of Michigan Medicine Pediatric Primary Care Group. These rural, suburban, and urban practice groups commit to enrolling their over 60 separate pediatric practice sites, each with an associated HTN subspecialist(s), and we will open enrollment to other private and non-university affiliated practice groups before the project begins, potentially broadening this cohort further. Additionally, the project will recruit through and is supported by the Midwest Pediatric Nephrology Consortium, a national organization with 73 pediatric member centers, multiple NIH grants, and over 30 ongoing multi-center studies, whose goal is to "improve and promote high quality care of pediatric nephrology patients through . . . collaborative clinical and translational research."[86] Nephrologists associated with the MWPNC will be able to identify practice groups who frequently refer to them, and suggest enrollment in this study. We ultimately consented 64 practices into the collaborative.

The main group of human subjects included in this research project are the adult physicians, nurses and office staff members whose behavior we will change to provide best-practice care for diagnosing and providing initial management for pediatric hypertension (HTN). We will collect pediatric patient HTN error data as a marker of this behavioral change but there will be no children directly involved in the research as study subjects. Please see attached consent form for each participating practice site. As this intervention is low to no-risk, represents standard of care and best practices that should be implemented regardless of this study, and data submitted to the research team is deidentified, we will request a waiver of consent for patient data access and have each practice site lead consent for their practice site's practitioners.

Of note, no patient names, medical record numbers, or dates of birth will be submitted to the project's REDCap database. The project does need to understand total days between clinic visits in order to know if time to definitive hypertension treatment has improved comparing intervention and control sites. The research team does not need to know specific clinic visit dates. In order to prevent undue burden on data entry staff at each clinic and institution, it is unreasonable to ask busy primary care pediatric clinic staff to repeatedly calculate the number of days elapsed between the initial clinic visit and multiple follow-up visits for multiple patients each month (see attached data entry form). For this reason, we will employ an "honest broker" for our REDCap database. Alexandre Peshansky, Lead Bioinformatics Analyst, Research Informatics Core (RIC), Albert Einstein College of Medicine; 1300 Morris Park Ave, Block Bldg., Rm 534 Bronx, NY 10461 (914) 294-6916. Mr. Peshansky has functioned in this role before and will create 2 separate REDCap databases. Participating clinics will have access to and enter data in a REDCap database that includes visit dates. The research team will never have access to this database. The second REDCap database, accessible by the research team, will receive days elapsed between clinic visit dates data from the

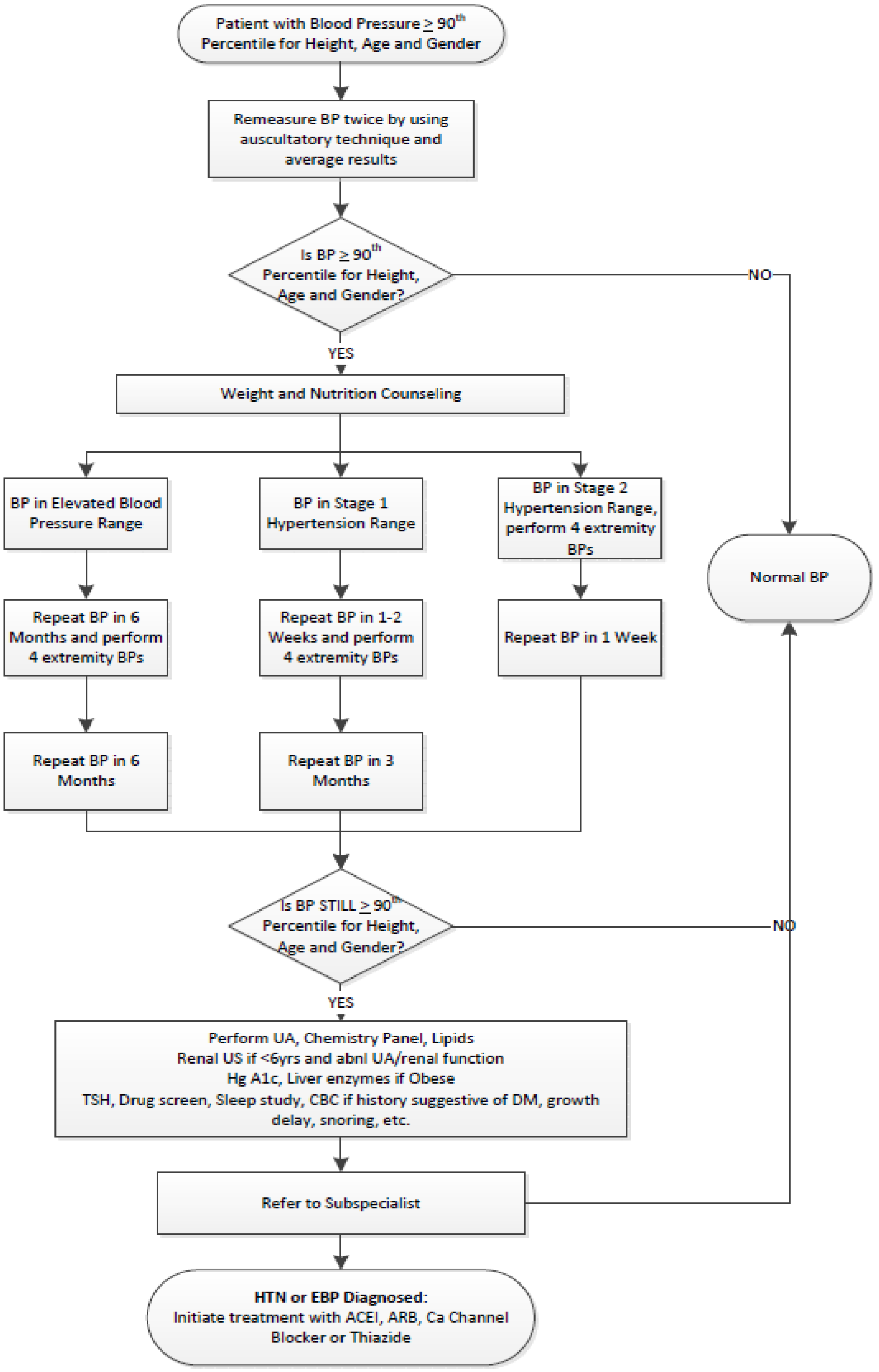

Figure 1: Steps to Diagnose Pediatric HTN and Initiate Definitive Management[6]

first database, but no specific clinic visits dates. Mr. Peshansky will ensure the research team never receives any PHI from clinics.

Table 1: Quality Improvement Collaborative (QIC) Components

• Multidisciplinary teams consisting of at least a physician, nurse and office practice associate<br>• Bi-annual 1-day interactive video webinar learning sessions<br>• Monthly all collaborative video webinar sharing best practices<br>• Monthly one on one team calls with dedicated QI coach (Research team)<br>• Monthly data submission using REDCap<br>• Monthly data feedback both at aggregate level with full inter-team transparency as well as at specific practice level<br>• Monthly mini-root cause analyses performed on 1 error at each site<br>• Instruction on best practices from content area experts in QI, and hypertension<br>• Instruction on model for improvement and behavior change via QI methodology (small tests of change/PDSA cycles)<br>• Instruction on QI team leadership and team QI skills<br>• Ongoing sharing of best ideas and barriers/issues among institutional teams

Cohort	PRE-WORK -3-0 Months	PHASE 1 0-6 Months	PHASE 2 7-12 Months	PHASE 3 13-18 Months	PHASE 4 19-24 Months
1	Pre-QIC data collection	QIC to Improve Local Hypertension Processes + Registry & BP Measurement	QIC with Subspecialist(s) to Improve Communication and Standardize Expectations	Hub and Spoke Co-Management QIC with Primary Care Hypertension Diagnosis and Management	Sustain Changes
2	Pre-QIC data collection	Usual Care + Registry & BP Measurement	QIC to Improve Local Hypertension Processes	QIC with Subspecialist(s) to Improve Communication and Standardize Expectations	Hub and Spoke Co-Management QIC with Primary Care Hypertension Diagnosis and Management

Figure 2: 28 month collaborative structure, Winter 2018-Spring 2020

The 6 committed practice groups have over 1,000 providers and 1.5 million pediatric patient visits annually. Each practice group, including additional ones not yet recruited, will receive approximately $5,000 annually to fund data collection processes. Each of the at least 60 practice sites will identify a multidisciplinary team consisting of at least one physician, nurse and practice associate, allowing for improvement across disciplines. Teams will be supported by a pre-identified relevant and local HTN subspecialist.

Similar to our current diagnostic error QIC, we will apply multivariate matching to practice groups before randomization, which has been shown to provide more accurate effect estimates, creating an equivalent of 7% increase in sample size.[87] Practice groups will be matched based on key demographics including 1) patient volume per practitioner +/- 100 patients, 2) patient population (rural, suburban or urban), 3) number of clinic sites, and 4) prior work reducing HTN diagnosis and management errors. These data as well as baseline demographic data and prior quality improvement experience data will be collected annually via the BP-CATCH Practice Inventory (attached). It will be completed by the lead practice physician following submission of a signed consent form to participate in the BP-CATCH project. The survey tool will be collected via SurveyMonkey, and administered at the start of the project, and twice annually thereafter. Similarly, we will administer a demographic survey to the hypertension subspecialists for each practice group. It will be completed by the hypertension subspecialist physician following submission of a signed consent form to participate in the BP-CATCH project. The survey tool will be collected via SurveyMonkey, and administered at the start of the project, and twice annually thereafter. Data from this survey will not be used in randomization. After matching, randomization will occur via computerized random number generation and practice groups will be assigned to one of 2 study cohorts.

Both cohorts will collect 3 months of retrospective baseline data collection before beginning the study. During the first six months of the intervention, the practice groups randomized to Cohort 1 will begin working on improving HTN practices within their clinic via a QIC, while the other cohort will act as a control (usual care) with data collection (**Specific Aim 1**). Cohort 1 will attend an initial 1-day interactive video webinar learning session where they will learn QI methodology, enhance and practice QI skills, identify local 30-60 day aims to improve local HTN practices and increase their understanding of pediatric HTN. They will also begin tracking data on a HTN registry and learn how to ensure accurate BP measurement is completed in their clinic. They will participate in QI coaching, monthly video conferences, and monthly mini-root cause analyses (Mini-RCAs)[91] (see below). Cohort

2 will submit control data and not receive centralized data feedback. They will also begin tracking data on a HTN registry and learn how to ensure accurate BP measurement is completed in their clinic.

During the second 6 months, Cohort 1 will integrate their HTN subspecialist into the QIC and focus on issues at the boundary of PCP and subspecialty care (e.g. pre-referral work-up, communication across providers, and time for next available appointment). Cohort 2 will begin improving HTN practices within their clinics via a separate but comparable PCP-only QIC, (**Specific Aim 2)**. In the third 6 months, Cohort 1 will continue QIC components and implement a hub and spoke model, where the PCP diagnoses and provides definitive management for pediatric HTN with subspecialist support and Cohort 2 will engage their HTN specialist (**Specific Aim 3**). In the final six months, Cohort 1 will sustain their changes, illustrating the durability of these system changes even after QIC completion. Cohort 2 will continue their QIC with the hub and spoke model.

This stepped wedge design allows for maximal improvement efforts across the largest number of practices while maintaining a rigorous, randomized design. Additionally it allows for the evaluation of the primary outcome comparisons (usual care versus local QIC without subspecialist versus QIC with subspecialist versus QIC with hub and spoke model) and secondary outcome comparisons: 1) change in collaborative effect for second wave participants (HTN error rates in Cohort 1 versus Cohort 2 during initial locally focused QICs), and 2) sustainability after hub and spoke model introduction (error rates in Cohort 1 in months 13-18 versus months 19-24).Figure 2. These secondary outcome comparisons will shed light on the effectiveness and sustainability of QICs and inform future QIC designs.

QIC intervention components closely mirror prior evidence on components integral to QIC success[42,43,47] and prior QICs conducted by the research team.[48,50,65] Prior research has shown that improving errors requires more than just EHR alerts,[88,89] which is why a QIC intervention was chosen. Important components of the QIC include rapid data feedback on performance with benchmarking, which is crucial to behavior change of clinicians.[90] The evidence-based Mini-RCAs[91] used in this and prior QICs, involve identifying an error monthly at each site, examining 15 standardized patient and systems factors that could have led to the error, and spreading systematic lessons learned to prevent future errors.[52,54] The QIC's interactive video webinar format was successfully employed in our diagnostic error QIC and allows for greater participation by providers who do not have time to travel and can engage more team members. The bundle of evidence-based tools implemented by the collaborative to help reduce HTN diagnosis and management errors will involve work from our prior QIC, and utilize concepts such as standardization, visual workflows, family engagement, EHR and handheld decision support, and streamlined communication.[8] As with any QIC, practice sites will adapt these tools, and develop new tools which will be quickly spread across the QIC to accelerate change and improve care delivery.

Finally, the pediatric hypertension specialist for each clinic group will track and report "time to 3rd next available appointment" for pediatric patients in their specialist clinic. This standard measure of patient access will help us understand if the hub and spoke model described above will reduce unneeded referrals to specialist clinics. The specialist will report this value on the first of every month during the baseline and intervention periods into REDCap.

Following the learning sessions in March of 2020, the study was put on hold. We are unable to continue with the original study design given the realities of pediatric care, telemedicine and the pressures put on clinics due to COVID-19 to focus on new care delivery systems that keep patients and staff safe. For that reason, we will ask a subset of already recruited Montefiore Medical Group practices to participate in a follow-up QI study examining the feasibility, acceptability and accuracy of home blood pressure monitoring (HBPM) measurements used for the confirmation of blood pressure elevation and diagnosis of hypertension. This strategy has the potential to provide a powerful new

tool for pediatricians to avoid repeated in-person BP measurements and speed time to pediatric hypertension diagnosis. We will analyze and publish data from the first year of the collaborative (November 2018-March 2020).

We will ask 9 already enrolled clinics if they are willing to participate in this new phase of the study. We will only study patients at 6 clinics, but because of the challenges of retaining sites during this pandemic, each clinic will be paired with another clinic at their site as a back-up. All clinics will be chosen from the 9 Montefiore Medical Group clinics that previously enrolled, allowing us to centralize the process for outreach and resulting). Sites will be given lists of 100 patients with one prior elevated BP measurement who were already included in data entered during the first phase of BP-CATCH (600 patients total). These patients will be 3 to 22 years old as this is the population to which the pediatric hypertension guidelines apply. The research coordinator will contact these 100 patients from each site and for 18-22 year olds, obtain verbal phone consent. For 7-17 year olds we will ask the caregiver to obtain verbal assent for participation. For 3-6 year olds, the caregiver will provide verbal consent. As our research assistant only speaks English, only English speaking participants will be enrolled in the study. Due to lower enrollment, we are increasing the number of patients we are calling from 50 to 100 and increasing from 5 to 6 sites. Each patient and caregiver will be requested to return to clinic to obtain a HBPM with an appropriately sized cuff based on arm circumference measurements, receive education on its use and instruction on how to record three morning and three evening blood pressures for seven days. In addition, each patient will undergo manual BP measurements to correlate with the BPs obtained by HBPM. This is a crucial and guideline based next clinical step, regardless of this study. We anticipate approximately 40 patients from each site will return to clinic to obtain the device (200 patients total). The study research coordinator will call patients one week later and ask for the BP measurements and ensure this information is sent to the appropriate clinician. We anticipate at least 20 patients from each site will return these data (100 patients total). These measurements will be entered into REDCap confidentially, along with dates of visit and measurements return. This will allow us to investigate the feasibility of HBPM as well as the correlation of HBPM with manual in-clinic BP measurements. Dates will be seen only by the REDCap “honest broker” and scrubbed from the database before being provided to the research team.

Patients over 7 years old with discordant BP categorization (e.g. normal, elevated, stage 1 or stage 2 hypertension) HBPM measurements and in-clinic BP measurements will be referred for a pediatric nephrology clinic appointment and loaned an ambulatory blood pressure monitoring device (ABPM), which is the gold standard for pediatric hypertension diagnosis. The ABPM will confirm either the HBPM or in-clinic measurement or neither. Since ABPM is not tolerated well for patients 3 to 7 years old, these patients will just be referred for a pediatric nephrology clinic appointment. In this manner, patients will receive hypertension diagnosis and more guideline compliant care. At the same time, we will be able to understand whether HBPM can be used an adjunct to in-clinic manual BP measurements.

4. Pediatric HTN Outcome Measures:

The research team has developed clearly defined primary and secondary outcome measures for pediatric HTN diagnosis and management errors, and has experience using evidence-based tools for their improvement. All outcome measures are described in Table 2**Error! Reference source not found.**. These measures will be compared between usual care, QIC without subspecialist involvement, QIC with subspecialist involvement, and hub and spoke QIC. The primary outcome is pediatric HTN or EBP diagnosis and management errors, defined as the number of patients with an elevated systolic or diastolic BP for age, sex and height (defined as $\geq$ 90th percentile for age, sex and height or >120/80, whichever is lower, in $\geq$ 3 year old patients) who did not have all correct diagnostic

and management decisions completed per 100 patients with an elevated BP measured across all 3 intervention conditions versus usual care (control) condition. Each practice site will identify the first 10 patients seen in their site monthly with an elevated BP and then evaluate those patients following the elevated BP to confirm correct HTN and EBP diagnosis and management steps. During the baseline period, this number will be 17 patients per month for 3 months, in order to provide an adequate baseline sample for practices to begin changing their practice. For example, if a patient already had one elevated BP reading in the Stage 1 HTN range and then presented for care, the practice site would record if they 1) received a repeat BP measurement in 1-2 weeks, 2) four extremity BP measurements and 3) a scheduled appointment for a repeat BP measurement in 3 months, etc… Similarly, if a patient already had 3 BPs in the Stage 1 HTN range and then presented to the practice site, this measure would track whether they received 1) all appropriate laboratory testing, 2) a subspecialist referral, and if diagnosed with HTN, 3) management with a recommended antihypertensive medication. In this way practice sites will be able to feasibly collect data, receive real-time and actionable data feedback, and work to reduce harmful errors. We acknowledge that HTN error measurement in the control period may introduce an intervention effect, but we have no reason to believe this effect will be different between the intervention and control practices.

As Secondary Outcome Measures, we will also track each of these markers of correct care individually and the time to completion from initial elevated BP measurement to each marker. This allows us to investigate how the different QIC conditions affect the proportion of patients who receive guideline recommended care, which diagnostic and management steps are correctly implemented, and the time it takes for patients to receive this care. We can then test error rate and time to care improvement between usual care, the QIC without subspecialist involvement, the QIC with subspecialist involvement, and the hub and spoke model QIC.

In order to confirm appropriate screening, practices will perform anonymous self-audits on the 5 most important aspects of BP measurement (a. choosing children to screen as per 2017 guideline recommendations[6]; b. positioning and calming a child for BP measurement; c. choosing an appropriately sized BP cuff; d. inflating the cuff to the appropriate pressure, and e. documenting percentiles and interpreting when the BP is elevated based on cumbersome age, sex and height tables) during the first 6 months of the project: either the initial local QIC without subspecialist involvement or during the baseline collection period for the control group. This anonymous self-audit will occur weekly for each person who shares primary responsibility for measuring pediatric blood pressures at triage. These data will be submitted anonymously into REDCap.

Finally, as noted above, we will compare time to 3rd next available appointment for pediatric patients to subspecialist clinics in order to assess the effects of pediatric primary care providers referring and managing different types of patients with hypertension.

Primary Outcome Measure	Number of patients without all correct diagnostic and management decisions (Figure 4) per 100 patients with measured elevated BP
Secondary Outcome Measures	1. Number of patients without correct diagnostic and management decisions at specified points per 100 patients with measured elevated BP<br>a. Re-measuring BP twice in clinic via auscultation<br>b. Weight and nutrition counseling<br>c. Repeat BP measurement visits appropriately timed<br>d. Initial laboratory workup<br>e. Subspecialist referral<br>f. Definitive medication management initiation<br>2. Mean time from first elevated BP to specific points in diagnostic and management process (see #1 above)<br>3. Number of BPs measured correctly with appropriately screened patient, patient position, cuff size, inflation, BP percentiles correctly documented and interpreted per 100 observations conducted<br>4. Time to 3rd next available subspecialist appointment

Table 2: Primary and Secondary Outcome Measures

5.

The primary outcome for the new phase of the study will be the feasibility and acceptance of HBPM by families of children with one elevated BP measurement. We will measure feasibility of HBPM by investigating 1) the percent of patients who were not interested in receiving the HBPM device, 2) the percent of patients who did not return any data on the HBPM measurements, 3) the percent of patients completing all requested measurements and the percent completing at least half

of the requested measurements, 4) the days between agreeing to come in for the device, to when they received the HBPM and when data was returned (actual dates will only be visible to the Honest Broker), and 4) the concordance between HBPM, and manual in-clinic BP measurements, and 5) percent of children diagnosed with hypertension. For those patients contacted for HBPM, we will continue to track the above pre-COVID outcomes as well. No data described above will be collected on patients not identified for this new phase of the study at this time.

DATA AND SAFETY MONITORING PLAN

The primary human subjects in this proposal are the adult physicians, nurses and office staff members whose behavior we will change to provide best-practice care for diagnosing and providing initial management for pediatric hypertension (HTN). We will collect pediatric patient HTN error data as a marker of this behavioral change but there will be no children directly involved in the research as study subjects. The project aims to aid clinicians in implementing best-practice, guideline recommended care around pediatric HTN. For this reason, this proposal is minimal to no-risk. Additionally, as practices will necessarily be aware of the quality improvement collaborative group they were randomized to, no data will be blinded. The proposal uses a stepped-wedge design, allowing all randomization cohorts to eventually receive the full intervention in order to meet clinical equipoise. All data will be fully transparent between quality improvement collaboratives to ensure maximal improvement on these standard-of-care measures. We have scheduled interim data analysis steps every six months of the project (6, 12 and 18 months) after the beginning of intervention, and our collaboratives will review data on a monthly basis in real time to ensure no unintended consequences of the project. Dr. Heo, Senior Biostatistician at the Albert Einstein College of Medicine, will ensure correct and statistically valid data analyses. Due to the minimal to no-risk nature of the study, the unblinded data collection, the standard of care of these interventions, and the real-time interim data analyses as part of the quality improvement collaborative, a formal Data and Safety Monitoring Board is not necessary. Oversight and monitoring to ensure the safety of participants and the validity and integrity of the data will be ensured by the entire research team, with particular focus by the PI and core leadership team: Drs. Rinke, Bundy and Brady.

Although unlikely given the standard nature of primary and secondary outcome measures in this project, if any practice sites are unable to collect data for any reason, they will be coached and attempts will be made to facilitate data collection. If they are still unable to collect data, the stopping rules at which time the practice will be asked to leave the project will include inability to collect data for 2 months following data coaching interaction. It is incredibly unlikely that any Adverse Events or Serious Adverse Events will occur as part of this standard of care study. If any do occur, or an Unanticipated Problem occurs, the PI, Dr. Rinke, will take full responsibility, immediately report the event to the research team, the lead-site's IRB (Einstein/Montefiore), the local site's IRB, the Agency for Healthcare Research and Quality, and other appropriate offices or agencies as needed.

Finally, all data from all sites will be entered into a REDCap data base. Data capture will be centralized via direct patient data entry or electronic data transfers into this REDCap database. REDCap is web-based and HIPAA compliant, and Dr. Rinke and Einstein/Montefiore will take responsibility for data security. These data will not include patient names, dates of birth, or medical record numbers. As noted above, we will employ an "honest broker" for our REDCap database with regards to clinic visit dates. Alexandre Peshansky has functioned in this role before and will create 2 separate REDCap databases. Participating clinics will have access to and enter data in a REDCap database that includes visit dates. The research team will never have access to this database. The second REDCap database, accessible by the research team, will receive days elapsed between clinic

visit dates data from the first database, but no specific clinic visits dates. Mr. Peshansky will ensure the research team never receives any PHI from clinics.

The grant supports an experienced REDCap data analyst at Montefiore/Einstein to create and manage these platforms and assist sites in data collection. REDCap is web-based and HIPAA compliant, and Dr. Rinke and Einstein/Montefiore will take responsibility for data security. REDCap will disseminate monthly feedback reports with full transparency and benchmarking to intervention practices. Practices and hypertension specialists will report demographic data annually, including patient volume, number and training level of providers, nursing and office staff, presence of trainees, and prior work aiming to improve HTN errors and QI context. Data collection sheets (see appendix) will assist practice teams in chart review and will mimic the REDCap database appearance.

Practice groups will work with the research team to create systems for standardized data collection and data entry. An orientation session will teach practices to identify each phase of pediatric HTN and EBP diagnosis and management, reliably collect these data via chart review and/or EHR data pulls and enter data into a centralized database. The research team will assist with issues and distribute clarifications to all teams to aid in standardization. As all 6 initially recruited practice groups employ integrated EHRs, practices will likely collect the above data by employing EHR based record review or data pulls, depending on abilities and interest of local EHRs. These tools will ensure standardized data collection by disparate practices and were pilot tested at MMG and the Johns Hopkins Harriet Lane Clinic. The Montefiore/Einstein-based project manager, closely supervised by Dr. Rinke, will ensure accurate and timely data submission by each practice site. Data will be checked for outliers or abnormal values and feedback given to practices to ensure consistency across practices. Site leads will perform secondary chart reviews to confirm data accuracy and aid in standardization. Dr. Rinke and this team successfully completed similar processes with 30 primary care pediatric practices for 24 months and 3 different pediatric errors without any funds to support obtaining data.

As noted above, each practice site will retrospectively identify the first 10 patients monthly with an elevated BP. Numbers of charts for review are based on power calculations and pragmatic considerations for busy PCPs. By selecting the first 10 patients with an elevated BP seen monthly, we ensure quasi-random selection of cases to review, while making data collection feasible. During the baseline period, this number will be 17 patients per month for 3 months, in order to provide an adequate baseline sample for practices to begin changing their practice. This pragmatic data collection will allow for greater generalizability of results and methods, and spread at the conclusion of the project. Each practice site will evaluate those patients following the elevated BP to confirm correct HTN and EBP diagnosis and management steps. Each step of the HTN diagnostic and management process will be checked and time stamped via standardized data collection sheets and/or EHR summary reports. As noted above, the research team will never have access to clinic visit dates, and these will be protected by Mr. Peshansky, who will serve as the honest broker for this project. All segments of the EHR will be searched to confirm diagnostic and management steps. If not already present by the eighth patient's chart reviewed each month, practices will specifically search for and collect data from at least two patients who proceeded through the diagnostic work-up phase until initiation of HTN management, to ensure data capture on the complete HTN diagnosis and management process. As 3-5% of patients have HTN,[1-4] this number of patients is obtainable in the primary care setting. Additionally, to ensure proper screening, all staff who routinely obtain patient BPs will complete a self- and/or observed audit form weekly on the 5 most important aspects of BP measurement during the QIC without subspecialist involvement until the site is consistently greater than 90% compliant. Finally, specialists will report time to 3rd next available appointment on the first of every month during the project's baseline and intervention periods.

For the 100 patients already identified by the study site as having an elevated BP measurement but no follow-up, data identified above will be collected. We will also collect data on manual in-clinic BP measurements, HBPM data, time between phone contact, receiving HBPM and returning data and ABPM data and nephrology follow-up data. All these data will be de-identified as described above and verbal phone consent will be obtained from caregivers and adult patients, and verbal assent will be obtained by the parent for children 7-17 years old. It is impractical for the study's one research assistant to be present at all 200 anticipated patient visits across 6 clinical sites to obtain in-person consent. Additionally, in the face of the COVID pandemic and the need to maintain social distancing, all MMG sites have restricted access to non-clinical research staff, especially in cases where consent and research processes can be conducted over the phone. Performing verbal consent will be safer for participants, the research assistant, and MMG staff in the involved sites. Additionally, it is challenging to obtain verbal assent from 7-17 year olds while on the phone, so we will ask the caregivers to perform that task under the research assistant's guidance. Paper versions of the completed consent form will be mailed to all participants. Providers and patients will be free to refuse to collect these data, refuse to receive the HBPM or the ABPM, or refuse to return to clinic as per normal care. See attached data collection form.

6. Statistical Analysis

<u>6.1 Statistical Models</u>:

The primary independent variable will be interventions with titrated QIC dosage levels stepped-up from the control condition (usual care) with scale scores from 0 (usual care) to 3 (hub and spoke). The primary dependent variable will be the proportion of all incorrect diagnostic and management decisions completed per 100 patients with elevated BPs. The individual-level outcome will be binary (incorrect or correct care), and absolute risk difference will be the primary measure of intervention effects. As there will be a multilevel hierarchy in the data structure, we will apply generalized mixed-effects models with identity link for testing the primary hypothesis in terms of risk difference. Site-specific, practice group-specific, and month-specific intercepts will be considered random to take into account potential variations in outcomes at all such levels in addition to correlations of individual-level outcome nested within each month for each practice. We will test if each step-up of the interventions (QIC without subspecialist, QIC with subspecialist, and hub and spoke) as compared to the control condition significantly reduce incorrect diagnosis and management proportions. We will also make all pairwise comparisons among the four conditions including usual care. All analyses will be controlled for potential confounding effects including factors such as baseline error rates, patient volume, number and training of providers, nursing and office staff, and prior work improving these errors.

For the secondary analyses testing 1) time to care, 2) sustainability, 3) change in collaborative effect for second wave and 4) necessity of PCP and subspecialist integration before hub and spoke model introduction, we will use all relevant QIC data. Each of these hypotheses will be tested with appropriate contrasts between intervention effect and the relevant group and time in generalized mixed-effects regression models. Testing the sustainability will be based on a contrast within Cohort 1.

The main outcome will be presented with descriptive statistics, examining the percent of patients who accepted the HBPM and returned data in a timely manner. We will also analyze the concordance between in-clinic manual BP measurements and the HBPM measurement averages.

<u>6.2 Data Entry Errors and Missing Data</u>:

We will make every effort to minimize data recording errors by checking for outliers or abnormal values using frequency tables or graphical tools such as box plots. If noted, we will correct or check

the validity of those values and discuss with sites. Although we expect few by virtue of randomization, we will examine imbalances between groups with respect to baseline practice demographic characteristics and outcome measurements with standard statistical methods depending on the outcome scale and validity of normal distribution assumptions. If necessary, we will apply log-transformations for right-skewed data. We will include variables that are significantly different in the above statistical models to control for their potential confounding effects. Although we anticipate little missing data based on prior QICs, when noted, we will examine reasons for incomplete records. We will analyze available data as the primary analytic strategy since mixed-effects modeling is valid for missing at random data. However, if missing data rate is greater than 5%, we will also conduct sensitivity analyses applying a full conditional specification multiple imputation method which can apply to missing not at random data. We will compare results between available data analysis and multiple imputations data analysis.

6.3 Power Analysis:

Assuming a two sided alpha level of 0.05 and >80% power, we calculated the effect size we could detect for the HTN and EBP diagnostic and management error primary outcome measure, comparing the three intervention conditions to the control condition and each other using the above models, and the QIC methodology and preliminary data (D.2.B.) presented in this application. As the data structure is complex with multilevel hierarchy in addition to introduction of titrated interventions in a stepped-wedge fashion, we conducted simulations to estimate empirical power to seek a minimally detectable effect size. We allowed substantial variations of the outcome at all levels of site, practice, and months. Specifically, we took 73% as the baseline diagnostic error rate under usual care and set the ranges of the outcome expectation as +/-15% at each level; for example, at the site level, the baseline incorrect diagnostic error rates will range from 73%-15% to 73%+15%, and so on. In addition, we set the outcome correlations among 10 chart reviews per month per site at a relatively large ICC=0.5. We used uniform distributions to randomly generate outcome expectations at all levels, and used Park's algorithm[92] to randomly generate individual chart-level correlated binary outcomes with ICC=0.5 for a given month. We generated 1000 simulation data sets for each candidate minimal effect size laid out in a grid search fashion and fitted the generalized mixed-effects model with random effect for all levels and counted p-values <0.05 out of 1000 for each effect size on the grid. As a result, our study with at least 7 practice groups, at least 60 sites, two cohorts, three intervention doses in addition to usual care, 24 months, and 10 chart reviews per month per practice is powered (>80%) to detect as small as 3% point decrease in each step-up 'titrated' dose, i.e., per each level increase in intervention dosage. Therefore, we are adequately powered to detect any decrease larger than 9% from usual care to hub and spoke condition.

Given the HTN error reduction estimates derived from our preliminary data and prior QIC, we are confident that we are powered to detect statistically and clinically significant effects in our primary outcome measure across all three intervention conditions.

As this new phase of BP-CATCH project will be a pilot and feasibility study related to whether HBPM can be used as a QI tool to reduce time to hypertension diagnosis, no pre-study power analysis will be conducted. We will calculate a post-hoc power analysis to determine the confidence intervals associated with the concordance between the HPBM and in-clinic manual BP measurements.

6.4. Limitations:

One limitation of this proposal is the potential variability in data collection methods in practice sites testing the QIC interventions to reduce HTN errors. The research team is experienced in running large, national QICs and ensuring data quality and timeliness across sites. Significant time at all learning sessions will be devoted to data acquisition and consistent outcome definitions, and practice

groups are receiving funds for data acquisition. A second limitation is that our practice groups are academic and interested in QI. They likely present a biased sample of pediatric practices. We believe this bias will improve the likelihood of success for the collaborative and will prove "what works" in pediatrics, further motivating late adopter practices. Finally, although there are other potentially more serious pediatric errors in terms of acute mortality, the HTN diagnostic and management errors were chosen because they are high frequency, generate high practitioner interest,[80] have clear recommendations for diagnosis, management and evidence-based interventions, and can serve as a model for other chronic care conditions.

6.5. Timeline for the Proposed Research and Deliverables:

This proposal will span three years, with IRB approval and preliminary QIC work including additional practice recruitment occurring in the first six months. While this timeline is compressed for QIC start-up, we are confident in our ability to complete it given our prior relationships with all practice groups, their advanced notice and interest in this collaborative, their prior QI work, and our prior QIC on elevated BP recognition which serves as a foundation for this broader effort. The QIC intervention will take place for two years, from the second half of year 1 through the first half of year 3. (Table 3) The PI and research group will provide AHRQ with an error toolkit for pediatric clinics and HTN subspecialists nationwide who may be interested in incorporating lessons learned to reduce HTN errors.

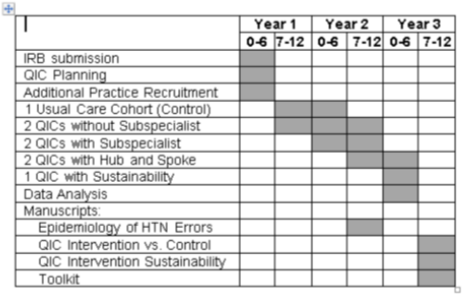

Table 3: Timeline for Research

7. Risks and Benefits:

<u>7.1 Potential Risks to Human Subjects</u>

The primary care physician, nurse and office associate health care providers at each of the practices, and their associated subspecialist, will be the primary human subjects for this work. As the proposal is geared toward improving providers' actions to be consistent with best-practice care, the risk to providers is minimal. No individual provider compliance data or diagnostic error data will be recorded. Only aggregated data at the practice level is recorded. There will be no additional medical risks in implementing these procedures as they are all standard of care and we are only working to ensure reliable implementation. Providers may feel discouragement if they are unable to reliably perform certain best-practices. In order to minimize this risk, we will provide constant encouragement, utilizing systems-focused, blame neutral coaching methodology. Parents and patients will be free to decline any aspect of care as occurs normally in care. As such, no additional risk is posed to the clinic patients. HBPM is safe, and no-risk. There will be no added risks to patients, families or providers due to this new addendum. HBPM is currently recommended for use in children with diagnosed hypertension, and for use in the diagnosis of pediatric hypertension by European medical guidelines.

As with any study, we will take significant steps to safeguard all personal health information collected. We will only collect the minimal amount of personal health information required as described above. All files will be de-identified and disassociated from practice identifiers as soon as the study is complete. All records will be kept in locked offices, on password protected computers and in password protected files. Finally, all data submitted to the REDCap database will be de-identified to the extent described above. Montefiore, using the REDCap software, has performed these activities, including the honest broker role by Mr. Peshansky, on multiple prior research projects. As such the risk of a breach of confidentiality is minimal and the legal risks associated with a breach of confidentiality are also to be minimal.

There are no foreseeable financial risks to patients as a result of this study. Providers could experience a potential decrease in patient volume and therefore financial revenue if their practice is worse than collaborative baseline and this information is made public. The research group will work hard to instill this understanding of confidentiality into the collaborative. On the contrary, practices could experience an increase in revenue if patients appreciate improved care and better diagnostic accuracy in their clinics. The financial risks to providers and practices are low.

7.2 Potential Benefits of the Proposed Research to Human Subjects and Others

The potential benefits of this study to the providers include timely and actionable feedback, and alerts to help providers better recognize aspects of diagnostic and management performance in need of improvement. Additionally, providers will be eligible to receive American Board of Pediatrics Part 4 Maintenance of Certification credit for each year of their participation. Although the subjects are the providers, pediatric patients may benefit from this study by being less likely to experience a HTN diagnosis or management error.

Each participating clinic will receive $3,000 as a data entry disbursement. Given the large number of BP measurements that must be entered into REDCap (at least 20 patients with 6 BP measurements each for 7 days = 840 measurements) this will subsidize the lost time to collect and enter these data. The disbursement will be disbursed when data entry is complete. Patients and families will benefit by receiving a return to clinic call for an elevated BP measurement, and a HBPM for their home use.

7.3 Importance of the Knowledge to be Gained

If this study is successful, it will provide data that best-practice care delivered via QICs can reduce HTN errors in ambulatory pediatric primary care patients. It will also suggest ways to move forward to reduce or eliminate errors for other pediatric chronic conditions and improve primary and subspecialty care interactions. Based on results of this study, hospitals and accreditors/regulators may consider tracking and reporting on HTN errors in primary care pediatrics. Given the minimal risks inherent in this proposal and the large potential benefit reducing these frequent pediatric HTN errors, we believe the risks are reasonable and are outweighed by the expected benefits. Similarly, if HBPM can speed diagnosis of pediatric hypertension, and prove an accurate tool to reduce the need for clinic visits, significant changes to pediatric hypertension diagnosis will be possible and patients will benefit.

8. Informed Consent

As noted above, six practice groups (Dartmouth Medical Center, Cleveland Clinic Children's, Johns Hopkins Harriet Lane Clinic, MUSC and SCPPRN, MMG and University of Michigan Medicine Pediatric Primary Care Group), with over 40 primary care practices have agreed to participate in this

important research effort. Additional practice groups will be enrolled via email list serves and word of mouth, attempting to enroll 60 practices at least, although we are adequately powered with the currently enrolled practice groups. Email listserv recruitment has been successful in prior and current quality improvement collaborative projects led by this investigative team. Practice groups will consent to participate via standardized consent forms, previously employed by this research team; see attached. As providers will experience minimal risk, only aggregate provider data will be reported, and the goal of this proposal is to ensure best-practices are completed by all providers. Similarly, because patients will experience minimal risk and only the minimal personal health information will be collected on patients, will obtain a waiver of consent for patient data.

For this new study, due to the need for standardizing data collection and entry, we will employ a single research coordinator for all sites. This research coordinator who is employed by Montefiore/Einstein, will contact all 600 eligible patients from the 6 Montefiore Medical Group Sites, inform them of the need for follow up blood pressure measurements, and will obtain verbal phone consent for the collection of home blood pressure monitoring data. While consent is not required for this clinical care intervention, verbal phone-based consent will be obtained for completeness. These patients will be 3 to 22 years old as this is the population to which the pediatric hypertension guidelines apply. The research coordinator will contact these 100 patients from each site and for 18-22 year olds, obtain verbal phone consent. For 7-17 year olds we will ask the caregiver to obtain verbal assent for participation. For 3-6 year olds, the caregiver will provide verbal consent. It is impractical for the study's one research assistant to be present at all 200 anticipated patient visits across 6 clinical sites to obtain in-person consent. Additionally, in the face of the COVID pandemic and the need to maintain social distancing, all MMG sites have restricted access to non-clinical research staff, especially in cases where consent and research processes can be conducted over the phone. Performing verbal consent will be safer for participants, the research assistant, and MMG staff in the involved sites. Paper versions of the completed consent form will be mailed to all participants. Additionally, it is challenging to obtain verbal assent from 7-17 year olds while on the phone, so we will ask the caregivers to perform that task under the research assistant's guidance. The HBPMs and the BP measuring process is low- to no-risk for patients and families, minimal data will be collected on patients, and no PHI will be seen by the research team, besides the research coordinator. Patients and parents will be free to decline the nephrology referral as with normal care.

9. Confidentiality

We will devote substantial effort and resources to protecting all participants from breaches of confidentiality. No patient or provider identifying information will be disclosed in reports, publications, or presentations. As noted above, all study personnel will complete required training in ethical procedures for the conduct of research. The investigators have extensive experience conducting HIPAA-concordant research and are regularly trained in the most current HIPAA regulations and procedures.

Any unanticipated problems will immediately be brought to the research team, and Einstein's and the relevant practice group's IRBs. As noted above, REDCap is a HIPAA compliant and previously tested data aggregation software. It is password protected, servers and data are kept in locked offices, and has not experienced a data breech in prior projects related to Montefiore. Only the research team will have access to the data. The research team and expert advisory panel will conduct interim analyses of collected data and qualitative reports from practices at 6, 12 and 18 months to ensure no unintended consequences of the project.

References:

1. Lurbe E, Alvarez J, Redon J. Diagnosis and treatment of hypertension in children. Current hypertension reports 2010;12:480-6.
2. Din-Dzietham R, Liu Y, Bielo MV, Shamsa F. High blood pressure trends in children and adolescents in national surveys, 1963 to 2002. Circulation 2007;116:1488-96.
3. Obarzanek E, Wu CO, Cutler JA, Kavey RE, Pearson GD, Daniels SR. Prevalence and incidence of hypertension in adolescent girls. The Journal of pediatrics 2010;157:461-7, 7 e1-5.
4. Muntner P, He J, Cutler JA, Wildman RP, Whelton PK. Trends in blood pressure among children and adolescents. JAMA 2004;291:2107-13.
5. Rosner B, Cook N, Portman R, Daniels S, Falkner B. Determination of blood pressure percentiles in normal-weight children: some methodological issues. Am J Epidemiol 2008;167:653-66.
6. Flynn JT, Kaelber DC, Baker-Smith CM, et al. Clinical Practice Guideline for Screening and Management of High Blood Pressure in Children and Adolescents. Pediatrics 2017.
7. Falkner B, Daniels SR. Summary of the Fourth Report on the Diagnosis, Evaluation, and Treatment of High Blood Pressure in Children and Adolescents. Hypertension 2004;44:387-8.
8. Brady TM, Neu AM, Siberry G, Solomon B. Increased Provider Recognition of Elevated Blood Presure in Children. American Society of Nephrology; 2012; San Diego, CA.
9. Hansen ML, Gunn PW, Kaelber DC. Underdiagnosis of hypertension in children and adolescents. JAMA 2007;298:874-9.
10. Bijlsma MW, Blufpand HN, Kaspers GJ, Bokenkamp A. Why pediatricians fail to diagnose hypertension: a multicenter survey. The Journal of pediatrics 2014;164:173-7 e7.
11. Sun SS, Grave GD, Siervogel RM, Pickoff AA, Arslanian SS, Daniels SR. Systolic blood pressure in childhood predicts hypertension and metabolic syndrome later in life. Pediatrics 2007;119:237-46.
12. Berenson GS, Srinivasan SR, Bao W, Newman WP, 3rd, Tracy RE, Wattigney WA. Association between multiple cardiovascular risk factors and atherosclerosis in children and young adults. The Bogalusa Heart Study. The New England journal of medicine 1998;338:1650-6.
13. Rademacher ER, Jacobs DR, Jr., Moran A, Steinberger J, Prineas RJ, Sinaiko A. Relation of blood pressure and body mass index during childhood to cardiovascular risk factor levels in young adults. Journal of hypertension 2009;27:1766-74.
14. Srinivasan SR, Myers L, Berenson GS. Changes in metabolic syndrome variables since childhood in prehypertensive and hypertensive subjects: the Bogalusa Heart Study. Hypertension 2006;48:33-9.
15. Urbina EM, Khoury PR, McCoy C, Daniels SR, Kimball TR, Dolan LM. Cardiac and vascular consequences of pre-hypertension in youth. J Clin Hypertens (Greenwich) 2011;13:332-42.
16. Tracy RE, Newman WP, 3rd, Wattigney WA, Berenson GS. Risk factors and atherosclerosis in youth autopsy findings of the Bogalusa Heart Study. Am J Med Sci 1995;310 Suppl 1:S37-41.
17. Donabedian A. The quality of care. How can it be assessed? JAMA 1988;260:1743-8.
18. Barnett ML, Song Z, Landon BE. Trends in physician referrals in the United States, 1999-2009. Archives of internal medicine 2012;172:163-70.
19. Pletcher BA, Rimsza ME, Cull WL, Shipman SA, Shugerman RP, O'Connor KG. Primary care pediatricians' satisfaction with subspecialty care, perceived supply, and barriers to care. The Journal of pediatrics 2010;156:1011-5, 5 e1.
20. Pediatric Specialist Physician Shortages Affect Access to Care. 2012. (Accessed August 31, 2017, at https://www.childrenshospitals.org/issues-and-advocacy/graduate-medical-education/fact-sheets/2012/pediatric-specialist-physician-shortages-affect-access-to-care.)
21. Stille CJ, Honigfeld L, Heitlinger LA, Kuo DZ, Werner EJ. The Pediatric Primary Care-Specialist Interface: A Call For Action. The Journal of pediatrics 2017;187:303-8.
22. Basco WT, Rimsza ME, Committee on Pediatric W, American Academy of P. Pediatrician workforce policy statement. Pediatrics 2013;132:390-7.

23. Yoon EY, Weber JS, McCool B, et al. Underlying Rationale and Approach to Treat Hypertension in Adolescents by Physicians of Different Specialty. Ann Pediatr Child Health 2013;1.
24. Stille CJ, Primack WA, McLaughlin TJ, Wasserman RC. Parents as information intermediaries between primary care and specialty physicians. Pediatrics 2007;120:1238-46.
25. Stille CJ, McLaughlin TJ, Primack WA, Mazor KM, Wasserman RC. Determinants and impact of generalist-specialist communication about pediatric outpatient referrals. Pediatrics 2006;118:1341-9.
26. Stille CJ, Primack WA, Savageau JA. Generalist-subspecialist communication for children with chronic conditions: a regional physician survey. Pediatrics 2003;112:1314-20.
27. Forrest CB, Glade GB, Baker AE, Bocian A, von Schrader S, Starfield B. Coordination of specialty referrals and physician satisfaction with referral care. Archives of pediatrics & adolescent medicine 2000;154:499-506.
28. Gandhi TK, Sittig DF, Franklin M, Sussman AJ, Fairchild DG, Bates DW. Communication breakdown in the outpatient referral process. Journal of general internal medicine 2000;15:626-31.
29. Ray KN, Ashcraft LE, Kahn JM, Mehrotra A, Miller E. Family Perspectives on High-Quality Pediatric Subspecialty Referrals. Acad Pediatr 2016;16:594-600.
30. Rubin K, Macary S, Cornell E, Chandhok L, Abebe Feyissa E, Honigfeld L. Working Together to Meet Children's Health Needs: Primary and Specialty Care Co-Management. Impact 2014.
31. Yoon EY, Kopec K, McCool B, et al. Differences in blood pressure monitoring for children and adolescents with hypertension among pediatric cardiologists and pediatric nephrologists. Clin Pediatr (Phila) 2014;53:1008-12.
32. Flynn JT, Alderman MH. Characteristics of children with primary hypertension seen at a referral center. Pediatr Nephrol 2005;20:961-6.
33. Baracco R, Kapur G, Mattoo T, et al. Prediction of primary vs secondary hypertension in children. J Clin Hypertens (Greenwich) 2012;14:316-21.
34. Flynn J, Zhang Y, Solar-Yohay S, Shi V. Clinical and demographic characteristics of children with hypertension. Hypertension 2012;60:1047-54.
35. Yoon E, McCool B, Filipp S, Rocchini A, Kershaw D, Clark S. Pediatric Hypertension Specialists' Perspectives About Adolescent Hypertension Management: Implications for Primary Care Providers. Clin Pediatr (Phila) 2015;54:551-6.
36. Specialist Physicians and Multidisciplinary Team Support Community-Based Primary Care Doctors, Enhancing Access to High-Quality HIV Care. 2011. (Accessed September 1, 2017, at https://innovations.ahrq.gov/profiles/specialist-physicians-and-multidisciplinary-team-support-community-based-primary-care.)
37. Chu C, Umanski G, Blank A, Grossberg R, Selwyn PA. HIV-infected patients and treatment outcomes: an equivalence study of community-located, primary care-based HIV treatment vs. hospital-based specialty care in the Bronx, New York. AIDS Care 2010;22:1522-9.
38. Hub and Spoke Initiative. 2015. (Accessed September 22, 2017, at https://www.aap.org/en-us/advocacy-and-policy/aap-health-initiatives/immunizations/Pages/Hub-and-Spoke-Initiative.aspx.)
39. Marcin JP, Nesbitt TS, Kallas HJ, Struve SN, Traugott CA, Dimand RJ. Use of telemedicine to provide pediatric critical care inpatient consultations to underserved rural Northern California. The Journal of pediatrics 2004;144:375-80.
40. Marcin JP, Ellis J, Mawis R, Nagrampa E, Nesbitt TS, Dimand RJ. Using telemedicine to provide pediatric subspecialty care to children with special health care needs in an underserved rural community. Pediatrics 2004;113:1-6.
41. Boneparth A, Flynn JT. Evaluation and treatment of hypertension in general pediatric practice. Clin Pediatr (Phila) 2009;48:44-9.
42. Schouten LM, Hulscher ME, van Everdingen JJ, Huijsman R, Grol RP. Evidence for the impact of quality improvement collaboratives: systematic review. Bmj 2008;336:1491-4.
43. Nadeem E, Olin SS, Hill LC, Hoagwood KE, Horwitz SM. Understanding the components of quality improvement collaboratives: a systematic literature review. The Milbank quarterly 2013;91:354-94.
44. Miller MR, Niedner MF, Huskins WC, et al. Reducing PICU central line-associated bloodstream infections: 3-year results. Pediatrics 2011;128:e1077-83.

45. Pronovost P, Needham D, Berenholtz S, et al. An intervention to decrease catheter-related bloodstream infections in the ICU. The New England journal of medicine 2006;355:2725-32.
46. Hinton CF, Neuspiel DR, Gubernick RS, et al. Improving newborn screening follow-up in pediatric practices: quality improvement innovation network. Pediatrics 2012;130:e669-75.
47. Hulscher ME, Schouten LM, Grol RP, Buchan H. Determinants of success of quality improvement collaboratives: what does the literature show? BMJ quality & safety 2013;22:19-31.
48. Rinke ML, Driscoll A, Mikat-Stevens N, et al. A Quality Improvement Collaborative to Improve Pediatric Primary Care Genetic Services. Pediatrics 2016;137:e20143874.
49. Bundy DG, Gaur AH, Billett AL, et al. Preventing CLABSIs among pediatric hematology/oncology inpatients: national collaborative results. Pediatrics 2014;134:e1678-85.
50. Rinke ML, Singh H, Heo M, et al. Diagnostic Errors in Primary Care Pediatrics: Project RedDE. Acad Pediatr 2017.
51. Rinke ML, Milstone AM, Chen AR, et al. Ambulatory pediatric oncology CLABSIs: epidemiology and risk factors. Pediatric blood & cancer 2013;60:1882-9.
52. Rinke ML, Chen AR, Bundy DG, et al. Implementation of a central line maintenance care bundle in hospitalized pediatric oncology patients. Pediatrics 2012;130:e996-e1004.
53. Rinke ML, Bundy DG, Milstone AM, et al. Bringing central line-associated bloodstream infection prevention home: CLABSI definitions and prevention policies in home health care agencies. Joint Commission journal on quality and patient safety / Joint Commission Resources 2013;39:361-70.
54. Rinke ML, Bundy DG, Chen AR, et al. Central line maintenance bundles and CLABSIs in ambulatory oncology patients. Pediatrics 2013;132:e1403-12.
55. Rinke ML, Zimmer KP, Lehmann CU, et al. Patient safety rounds in a pediatric tertiary care center. Joint Commission journal on quality and patient safety / Joint Commission Resources 2008;34:5-12.
56. Rinke ML, Shore AD, Morlock L, Hicks RW, Miller MR. Characteristics of pediatric chemotherapy medication errors in a national error reporting database. Cancer 2007;110:186-95.
57. Rinke ML, Moon M, Clark JS, Mudd S, Miller MR. Prescribing errors in a pediatric emergency department. Pediatric emergency care 2008;24:1-8.
58. Rinke ML, Dietrich E, Kodeck T, Westcoat K. Operation care: a pilot case management intervention for frequent emergency medical system users. The American journal of emergency medicine 2012;30:352-7.
59. Rinke ML, Bundy DG, Shore AD, Colantuoni E, Morlock LL, Miller MR. Pediatric antidepressant medication errors in a national error reporting database. Journal of developmental and behavioral pediatrics : JDBP 2010;31:129-36.
60. Rinke ML, Bundy DG, Abdullah F, Colantuoni E, Zhang Y, Miller MR. State-Mandated Hospital Infection Reporting Is Not Associated With Decreased Pediatric Health Care-Associated Infections. J Patient Saf 2014.
61. Tarini BA, Brooks MA, Bundy DG. A policy impact analysis of the mandatory NCAA sickle cell trait screening program. Health Serv Res 2012;47:446-61.
62. Strouse JJ, Reller ME, Bundy DG, et al. Severe pandemic H1N1 and seasonal influenza in children and young adults with sickle cell disease. Blood 2010;116:3431-4.
63. Bundy DG, Strouse JJ, Casella JF, Miller MR. Urgency of emergency department visits by children with sickle cell disease: a comparison of 3 chronic conditions. Academic pediatrics 2011;11:333-41.
64. Bundy DG, Muschelli J, Clemens GD, et al. Ambulatory care connections of Medicaid-insured children with sickle cell disease. Pediatric blood & cancer 2012;59:888-94.
65. Gaur AH, Bundy DG, Gao C, et al. Surveillance of hospital-acquired central line-associated bloodstream infections in pediatric hematology-oncology patients: lessons learned, challenges ahead. Infection control and hospital epidemiology : the official journal of the Society of Hospital Epidemiologists of America 2013;34:316-20.
66. Bundy DG, Persing NM, Solomon BS, et al. Improving immunization delivery using an electronic health record: the ImmProve project. Academic pediatrics 2013;13:458-65.
67. Pruette CS, Fivush BA, Flynn JT, Brady TM. Effects of obesity and race on left ventricular geometry in hypertensive children. Pediatr Nephrol 2013;28:2015-22.
68. Brady TM, Redwine KM, Flynn JT, American Society of Pediatric N. Screening blood pressure measurement in children: are we saving lives? Pediatr Nephrol 2014;29:947-50.

69. Brady TM. The Role of Obesity in the Development of Left Ventricular Hypertrophy Among Children and Adolescents. Current hypertension reports 2016;18:3.
70. Brady TM. Poor elevated-blood-pressure recognition in the outpatient setting. The Journal of pediatrics 2015;167:780.
71. Brady TM. Hypertension. Pediatr Rev 2012;33:541-52.
72. Misurac JM, VanDeVoorde RG, Kallash M, et al. Immunogenicity of Augmented Compared With Standard Dose Hepatitis B Vaccine in Pediatric Patients on Dialysis: a Midwest Pediatric Nephrology Consortium Study. Clin J Am Soc Nephrol 2017;12:772-8.
73. Wiesen J, Adkins M, Fortune S, et al. Evaluation of pediatric patients with mild-to-moderate hypertension: yield of diagnostic testing. Pediatrics 2008;122:e988-93.
74. Boyden LM, Choi M, Choate KA, et al. Mutations in kelch-like 3 and cullin 3 cause hypertension and electrolyte abnormalities. Nature 2012;482:98-102.
75. Goilav B, Satlin LM, Wilson PD. Pathways of apoptosis in human autosomal recessive and autosomal dominant polycystic kidney diseases. Pediatr Nephrol 2008;23:1473-82.
76. Goilav B, Putterman C, Rubinstein TB. Biomarkers for kidney involvement in pediatric lupus. Biomark Med 2015;9:529-43.
77. Goilav B, Putterman C. The Role of Anti-DNA Antibodies in the Development of Lupus Nephritis: A Complementary, or Alternative, Viewpoint? Semin Nephrol 2015;35:439-43.
78. Goilav B, Norton KI, Satlin LM, et al. Predominant extrahepatic biliary disease in autosomal recessive polycystic kidney disease: a new association. Pediatr Transplant 2006;10:294-8.
79. Goilav B. Apoptosis in polycystic kidney disease. Biochim Biophys Acta 2011;1812:1272-80.
80. Rinke ML, Singh H, Ruberman S, et al. Primary Care Pediatricians' Interest in Diagnostic Error Reduction. Diagnosis 2016.
81. Heo M, Kim N, Rinke ML, Wylie-Rosett J. Sample size determinations for stepped-wedge clinical trials from a three-level data hierarchy perspective. Stat Methods Med Res 2016.
82. Bundy DG, Rinke ML, Shore AD, Hicks RW, Morlock LL, Miller MR. Medication errors in the ambulatory treatment of pediatric attention deficit hyperactivity disorder. Joint Commission journal on quality and patient safety / Joint Commission Resources 2008;34:552-9, 497.
83. Rinke ML, Zimmer KP, Lehmann CU, et al. Patient safety rounds in a pediatric tertiary care center. Joint Commission journal on quality and patient safety / Joint Commission Resources 2008;34:5-12.
84. Pronovost PJ, Berenholtz SM, Needham DM. Translating evidence into practice: a model for large scale knowledge translation. Bmj 2008;337:a1714.
85. Brady TM, Neu AM, Miller ER, 3rd, Appel LJ, Siberry GK, Solomon BS. Real-time electronic medical record alerts increase high blood pressure recognition in children. Clin Pediatr (Phila) 2015;54:667-75.
86. Midwest Pediatric Nephrology Consortium Mission. 2017. (Accessed September 1, 2017, at http://mwpnc.com/about.)
87. Greevy R, Lu B, Silber JH, Rosenbaum P. Optimal multivariate matching before randomization. Biostatistics 2004;5:263-75.
88. Sittig DF, Teich JM, Osheroff JA, Singh H. Improving clinical quality indicators through electronic health records: it takes more than just a reminder. Pediatrics 2009;124:375-7.
89. Singh H, Arora HS, Vij MS, Rao R, Khan MM, Petersen LA. Communication outcomes of critical imaging results in a computerized notification system. Journal of the American Medical Informatics Association : JAMIA 2007;14:459-66.
90. Berner ES, Graber ML. Overconfidence as a cause of diagnostic error in medicine. The American journal of medicine 2008;121:S2-23.
91. Giardina TD, King BJ, Ignaczak AP, et al. Root cause analysis reports help identify common factors in delayed diagnosis and treatment of outpatients. Health affairs 2013;32:1368-75.
92. Park CG, Park T, Shin DW. A simple method for generating correlated binary variates. American Statistician 1996;50:306-10.

APPENDIX:

BP-CATCH DATA COLLECTION GUIDANCE
Version 1.3 10/19/18

Chart Pull for Eligible Patients:

- Select healthcare maintenance visits or non-acute care visits* of patients age 3 years through 21 years old.
- Identify the first 10 patient charts (or 17 in the baseline period) that meet inclusion criteria (see below) each month. Start with patients seen in the morning on the 1st of the month, and move forward until 10 patients have been identified. Data from these patient charts will be entered into the REDCap data collection form. Your clinic will retain the Protected Health Information in a separate location so that data from subsequent visits can be longitudinally entered and tracked as needed.

*Initial visit chart pull occurs for healthcare maintenance visits or non-acute care visits only. Subsequently, those same patients, when seen for any reason, may be reviewed for chart review if follow-up is needed.

Inclusion Criteria for Initial Visit:

- A patient age $\geq$ 3 years old and $\leq$21 years old
- Blood pressure (BP) is elevated $\geq$ 90th percentile for patient's height, weight and sex, or $\geq$120/80 (regardless of height/sex/weight) at a healthcare maintenance visit or non-acute care visit (e.g. chronic disease follow-up visit). NOTE: This elevated BP measurement can result from oscillometry or auscultation; and it meets inclusion criteria even if a repeated measurement at that visit is normal.

Exclusion Criteria for Initial Visit:

- Prior hypertension/elevated BP diagnosis. NOTE: Patient can have prior elevated BP measurements as long as no diagnosis has been made
- BP$\geq$95th percentile + 30mm or >180/120 or symptomatic patient
- Prior diagnosis of congenital heart disease, chronic kidney disease, urologic disease (e.g. posterior urethral valve, vesicoureteral reflux) or organ transplant,
- Previously included in BP-CATCH data entry
- Acute care visit (e.g., fever, viral illness, asthma attack, pain in any body part, etc.)

Protected Health Information to be held by the Practice:

These data below are included in the data collection form below for ease of use by the practices, but will NOT be entered into REDCap, nor collected by the collaborative. The practice, and particularly the team member entering the patient data each month, will decide how and where to retain the following information locally for subsequent data pulls:

- Patient Last Name
- Patient First Name
- MRN
- Date of birth

Suggestions for retaining this information at your practice:

- Electronic options:
    - Use a password protected Excel spreadsheet which has a unique identifier (e.g., alpha numeric, random assignment) assigned to each patient. Include the above four pieces of information in that Excel. A blank version of this Excel sheet can be provided on request from the BP-CATCH leadership team.

    OR

    - Utilize registry functionality in your EHR.
- Separately track the visits of patients with unique identifiers for each visit.
- Keep printed copies of the chart review tool forms in locked cabinets.

Protected Health Information to be held by an "Honest Broker":
Our project team needs to know how many days elapsed between certain dates (e.g. number of days between initial visit and sending laboratory studies). In order to avoid making each practice count the number of days elapsed between the initial visit date and other dates (could be over 400 days), and to avoid submitting PHI to the research team, we are using an IRB approved "Honest Broker" technique. This means that practices will enter specific visit dates into REDCap, but these dates will NEVER be given to the research team and will be kept in a separate database. The research team will only have access to days elapsed between visits which is provided to them from the Honest Broker. The Honest Broker for this project is Alexandre Peshansky, Lead Bioinformatics Analyst, Research Informatics Core (RIC), Albert Einstein College of Medicine; 1300 Morris Park Ave, Block Bldg., Rm 534 Bronx, NY 10461 (914) 294-6916.

Important Definitions:

- **Manual Blood Pressure:** auscultatory measurements taken with a stethoscope.
- **Years:** As it refers to age, round to nearest half year (i.e., patient that is 4 years, 3 months old would be charted as 4.5 years old; patient that is 9 years, 2 months old would be charted as 9 years old).
- **How many elevated BPs did the patient have at unique visits?:** In last 24 months, how many visits (healthcare maintenance or non-acute) did the patient have elevated BPs? A unique visit occurs on separate days. e.g. If patient had an elevated BP on Monday 12 months prior and came back on Tuesday 12 months prior and also had an elevated BP and neither visit include a normal BP at THAT visit, this patient would have 2 prior unique visits with an elevated BP. "This visit" would be the patient's "Third Elevated BP."
- **BP in Elevated Blood Pressure Range:** ≥90th to <95th Percentiles or 120/<80 to 129/<80
- **BP in Stage I Hypertension Range:** ≥95th to <95th + 12mm or 130/80 to 139/89
- **BP in Stage II Hypertension Range:** ≥95th+12mm to <95th+30mm or ≥140/90 to <180/120
- **BP warranting immediate referral**: ≥95th + 30 mmHg or ≥180/120 or patient with symptoms
- **Subspecialist**: Nephrologist or Cardiologist.
- "No" means the same as "**Not Documented"**
- **Was a referral request to a subspecialist made?**: did the PCP or other office staff tell the patient or write a specific note for the patient suggesting they need to go see a subspecialist provider?
- **Date the referral request to a subspecialist was made**: The date the provider or other person told/wrote that the patient should go to the subspecialist is the "date" the referral was made. This is not the appointment date that the patient may have received. This is not the date the patient actually went to the subspecialist.

- **Date of first specialist visit completed:** date patient saw a subspecialist for the first time. NOTE: Only applies to completed visits; scheduled visits which result in a no-show do not count.
- **Weight counseling**: discussion of the need to lose weight
- **Nutrition counseling:** discussion of healthy foods and/or need to eat less salt
- **Lifestyle modifications:** move more, less screen time, etc.
- **Abnormal extremity blood pressures:** any leg BP same or lower than arm BP
- **"Relevant" BP laboratory evaluations:** include CBC, Chemistry Panel (Cr, BUN, electrolytes), Urine Analysis, Fasting Serum Glucose, Lipids, AST/ALT, HgA1c, TSH, or Drug screen. These must be sent to evaluate blood pressure elevations, and not for other reason (e.g. sending a urine analysis for concern of a UTI)
- **"Relevant" radiologic or other evaluations:** include Renal US, Sleep Study, CTA, or MRA. These must be sent to evaluate blood pressure elevations, and not for another reason

Please reach out to the BP-CATCH expert group with all questions or concerns: Dr. Michael Rinke mrinke@montefiore.org 718-741-2597

Patient Information: **FOR CLINIC USE ONLY; NOT ENTERED INTO REDCap**

1. Patient Last Name: ___________________________
2. Patient First Name: ___________________________
3. MRN: _________________________________
4. Date of Birth (MM/DD/YYYY): _ _ /_ _ / _ _ _ _
5. Patient's chart FIRST reviewed on (MM/DD/YYYY): _ _ /_ _ / _ _ _ _
6. REDCap Record ID: _____

EXCLUDE Patients: not at healthcare maintenance visits or non-acute care visits, prior hypertension/elevated BP diagnosis, prior diagnosis of congenital heart disease, Chronic Kidney Disease, urologic disease, or organ transplant, BP≥95th percentile + 30mm or >180/120 or symptomatic patient, previously included in BP-CATCH data entry

A. Patient Demographic Information

1. Patient age (in years) at **this visit**: __________________ years (round to nearest 0.5 year)

2. Patient's Race: ☐ White ☐ Black ☐ Asian ☐ Native American/Alaskan Native ☐ Multiple Races ☐ Unknown

3. Patient's Ethnicity: ☐ Not Hispanic ☐ Hispanic ☐ Unknown

4. Patient's Insurance Status: ☐ Private ☐ Public ☐ Uninsured/Self Pay ☐ Unknown

5. Patient's Sex: ☐ Male ☐ Female

B. How Many Elevated BPs at Unique Visits in the Last 24 Months?

1. In the last 24 months, at healthcare maintenance and/or non-acute care visits, how many elevated BPs did the patient have at unique visits? Please count total number of unique visits with abnormal BPs and no normal BPs at those visits in the last 24 months at a healthcare maintenance or non-acute care visits and check appropriate box

***Note**: A patient may also have one or more normal BPs at separate unique visits in the last 24 months at healthcare maintenance or non-acute care visits and be included.*

☐ This visit is their **First elevated BP** [Go to Section C]

☐ This visit is their **Second elevated BP** [Go to Section D]

☐ This visit is their **Third elevated BP** [Go to Section E]

☐ This visit is their **Fourth or more elevated BPs** [Go to Section E]

☐ This visit is their first visit to this clinic or in our EHR. Therefore, this visit is their **First elevated BP** [Go to Section C]

C. **First Elevated BP** in last 24 months at healthcare maintenance or non-acute care visit

1. Date of visit with first elevated BP noted (MM/DD/YYYY): _ _ /_ _ / _ _ _ _ [THESE DATA ARE ENTERED INTO REDCAP BUT NOT GIVEN TO RESEARCH TEAM]

   1a. Height at **this visit with an elevated blood pressure** _____ (centimeters) ☐Not done at this visit

   1b. Weight at **this visit with an elevated blood pressure** _____ (kilograms) ☐Not done at this visit

2. Initial Systolic Blood Pressure: _______________ Systolic Percentile: ____________ ☐ Percentile unknown

3. Initial Diastolic Blood Pressure: _______________ Diastolic Percentile: ___________ ☐ Percentile unknown

4. Blood pressure measured by: ☐ Automated (Oscillometry) [Go to 4a] ☐ Manual (Auscultation) [Go to 4d]

   4a. If Automated: was BP remeasured twice with automated device? ☐ Yes [Go to 4a.i] ☐ No [Go to 4c]

      4a.i. If Yes, what were 2 additional results: Systolic Blood Pressure #2: _______________
      Diastolic Blood Pressure #2: _______________

Systolic Blood Pressure #3: _______________
Diastolic Blood Pressure #3: _______________

[Go to 4b]

4b. Was average of 2 repeat automated BP still elevated (≥ 90th percentile for patient's height, weight and sex, or ≥120/80)?

☐ Yes [Go to 4d] ☐ No [**STOP COMPLETING FORM**]

4c. Was repeat automated BP measured only one more time? (i.e. it was not remeasured twice and averaged)

☐ Yes and it was normal [**STOP COMPLETING FORM**]

☐ Yes and it was still elevated (≥ 90th percentile for patient's height, weight and sex, or ≥120/80) [Go to 4d]

☐ No, BP Not re-measured [Go to 5]

4d. If first Manual was abnormal, or repeat automated BPs still elevated: was BP remeasured twice with manual ?

☐ Yes [Go to 4d.i] ☐ No [Go to 4e]

4d.i. If Yes, what were 2 additional results: Systolic Blood Pressure #2: _______________
Diastolic Blood Pressure #2: _______________
Systolic Blood Pressure #3: _______________
Diastolic Blood Pressure #3: _______________

4d.ii.Was average of 2 Manual BPs still elevated (≥ 90th percentile for patient's height, weight and sex, or ≥120/80)?

☐ Yes [Go to 5] ☐ No [**STOP COMPLETING FORM**]

4e. Was repeat Manual BP measured only one more time? (i.e. it was not remeasured twice and averaged)

☐ Yes and it was normal [**STOP COMPLETING FORM**]

☐ Yes and it was still elevated (≥ 90th percentile for patient's height, weight and sex, or ≥120/80) [Go to 5]

☐ No, BP Not re-measured [Go to 5]

5. Did provider document that the following were discussed with patient and/or family:

5a. Weight counseling ☐ Yes ☐ No ☐ N/A patient has normal BMI

5b. Nutrition counseling ☐ Yes ☐ No

5c. Lifestyle modifications ☐ Yes ☐ No

6. Did the patient have BP in Stage II Hypertension range (≥95th+12mm to <95th+30mm; or ≥140/90 to <180/120)?

☐ Yes [Go to 6a] ☐ No [Go to 7]

6a. If patient had BP in Stage II Hypertension range (≥95th+12mm or ≥140/90) were both upper extremities and 1 lower extremity blood pressure taken and documented?

☐ Yes [Got to 6b] ☐ No [Go to 7]

6b. If yes, were they normal? ☐ Yes [Go to 7] ☐ No [Go to 6c]

6c. If abnormal, was a referral request to a subspecialist made? ☐ Yes [Got to 6d] ☐ No [Go to 7]

*Did someone in the clinic tell the patient/family they needed to see a subspecialist and complete referral paperwork?

6d. If a subspecialist referral was made, which specialist? (Check all that apply):

☐Nephrologist ☐Cardiologist ☐ Other: ____

6e. ☐N/A: Patient never attended subspecialist visit [Go to 7]

[IF N/A NOT CHECKED] Date first subspecialist visit <u>completed</u> (MM/DD/YYYY): _ _ /_ _ / _ _ _ _ [THESE DATA ARE ENTERED INTO REDCAP BUT NOT GIVEN TO RESEARCH TEAM]

*<u>Not date referral request made</u>, but date patient actually saw subspecialist

6f. Date information on the patient was received from subspecialist back to the pediatrician:

(MM/DD/YYYY): _ _ /_ _ / _ _ _ _ [THESE DATA ARE ENTERED INTO REDCAP BUT NOT GIVEN TO RESEARCH TEAM]

*If same electronic health record, enter date note was signed by the subspecialist

7. Was a follow-up appointment recommended or made for the patient with the primary care practice?

☐ Yes [Go to 7a] ☐ No [Go to Section D]

7a. If yes, what date was the follow-up appointment scheduled for? (MM/DD/YYYY): _ _ /_ _ / _ _ _ _ [THESE DATA ARE ENTERED INTO REDCAP BUT NOT GIVEN TO RESEARCH TEAM]

D. Second Elevated BP

If this is the initial visit which qualifies a patient for BP-CATCH, this <u>must</u> be a healthcare maintenance or non-acute care visit. If this patient previously had a qualifying BP-CATCH elevated BP visit above, then this can be any visit when the patient returned for BP follow-up.

1. Date of visit with second elevated BP noted (MM/DD/YYYY): _ _ /_ _ / _ _ _ _ [THESE DATA ARE ENTERED INTO REDCAP BUT NOT GIVEN TO RESEARCH TEAM]

1a. Height at **<u>this visit with an elevated blood pressure</u>** _____ (centimeters) ☐Not done at this visit

1b. Weight at **<u>this visit with an elevated blood pressure</u>** _____ (kilograms) ☐Not done at this visit

2. Systolic Blood Pressure: ______________ Systolic Percentile: ___________ ☐ Percentile unknown

3. Diastolic Blood Pressure: ______________ Diastolic Percentile: __________ ☐ Percentile unknown

4. Blood pressure measured by: ☐ Automated (Oscillometry) [Go to 4a] ☐ Manual (Auscultation) [Go to 4c]

4a. Was this BP repeated by <u>manual</u> (auscultation)? ☐ Yes [Go to 4b] ☐ No [Go to 4a.i.]

4a.i. Was <u>automated</u> BP still elevated (≥ 90th percentile for patient's height, weight and sex, or ≥120/80)?

☐ No [**STOP COMPLETING FORM**]

☐ Yes (≥ 90th percentile for patient's height, weight and sex, or ≥120/80) [Go to 5]

4b. What were <u>manual</u> BP results: Systolic Blood Pressure: ______________
Diastolic Blood Pressure: ______________

4c. Was BP re-measured again <u>with manual</u> ?

☐ Yes [Go to 4c.i] ☐ No [Go to 4e]

4c.i. If Yes, what were <u>additional</u> results: Systolic Blood Pressure #2: ______________

Diastolic Blood Pressure #2: _______________ [Go to 4d]

4d. Was average of 2 manual BPs still elevated (≥ 90th percentile for patient's height, weight and sex, or ≥120/80)?

☐ Yes [Go to 5] ☐ No [**STOP COMPLETING FORM**]

4e. Was first manual BP elevated (≥ 90th percentile for patient's height, weight and sex, or ≥120/80)?

☐ Yes [Go to 5] ☐ No [**STOP COMPLETING FORM**]

5. Did provider document that the following were discussed with patient and/or family:

5a. Weight counseling ☐ Yes ☐ No ☐ N/a patient has normal BMI

5b. Nutrition counseling ☐ Yes ☐ No

5c. Lifestyle modifications ☐ Yes ☐ No

6. Did the patient have BP in Stage II Hypertension range (≥95th+12mm to <95th+30mm; or ≥140/90 to <180/120)?

☐ Yes [Go to 6a] ☐ No [Go to 7]

6a. Did the patient have BP in Stage II Hypertension range (≥95th+12mm to <95th+30mm; or ≥140/90 to <180/120) at the prior visit?

☐ Yes [Go to 6b] ☐ No [Go to 7]

6b. Did the provider document a diagnosis of 'Stage II Hypertension' in notes, billing or problem lists at this visit?

☐ Yes [Go to Section F] ☐ No [Go to Section F]

7. Were both upper extremities and 1 lower extremity blood pressure taken and documented?

☐ Yes [Got to 7a] ☐ No [Go to 8]

7a. If yes, were they abnormal (any leg BP same or lower than arm BP)? ☐ Yes [Go to 7b] ☐ No [Go to 8]

7b. If abnormal, was a referral request to a subspecialist made? ☐ Yes [Go to 7c]☐ No [Go to 8]

*Did someone in the clinic tell the patient/family they needed to see a subspecialist and complete referral paperwork?

7c. If a subspecialist referral was made, which specialist? (Check all that apply):

☐Nephrologist ☐Cardiologist ☐ Other: ____

7d. ☐N/A: Patient never attended subspecialist visit [Go to 8]

[IF N/A NOT CHECKED] Date first subspecialist visit completed (MM/DD/YYYY): _ _ /_ _ / _ _ _ _ [THESE DATA ARE ENTERED INTO REDCAP BUT NOT GIVEN TO RESEARCH TEAM]

*Not date referral request made, but date patient actually saw subspecialist

7e. Date information on the patient was received from subspecialist back to the pediatrician: (MM/DD/YYYY): _ _ /_ _ / _ _ _ _ [THESE DATA ARE ENTERED INTO REDCAP BUT NOT GIVEN TO RESEARCH TEAM]

*If same electronic health record, enter date note was signed by the subspecialist

8. Was a follow-up appointment recommended or made for the patient with the primary care practice?

☐ Yes [Go to 8a] ☐ No [Go to Section E]

8a. If yes, what date was the follow-up appointment scheduled for? (MM/DD/YYYY): _ _ /_ _ / _ _ _ _ [THESE DATA ARE ENTERED INTO REDCAP BUT NOT GIVEN TO RESEARCH TEAM]

E. Third Elevated BP in last 24 months at healthcare maintenance or non-acute care visit

If this is the initial visit which qualifies a patient for BP-CATCH, this <u>must</u> be a healthcare maintenance or non-acute care visit. If this patient previously had a qualifying BP-CATCH elevated BP visit above, then this can be any visit when the patient returned for BP follow-up.

1. Date of visit with third or more elevated BP noted (MM/DD/YYYY): _ _ /_ _ / _ _ _ _ [THESE DATA ARE ENTERED INTO REDCAP BUT NOT GIVEN TO RESEARCH TEAM]

1a. Height at **<u>this visit with an elevated blood pressure</u>** _____ (centimeters) ☐Not done at this visit

1b. Weight at **<u>this visit with an elevated blood pressure</u>** _____ (kilograms) ☐Not done at this visit

2. Systolic Blood Pressure: ______________ Systolic Percentile: ___________ ☐ Percentile unknown

3. Diastolic Blood Pressure: ______________ Diastolic Percentile: __________ ☐ Percentile unknown

4. Blood pressure measured by: ☐ Automated (Oscillometry) [Go to 4a] ☐ Manual (Auscultation) [Go to 4c]

4a. Was this BP repeated by <u>manual</u> (auscultation)? ☐ Yes [Go to 4b] ☐ No [Go to 4a.i.]

4a.i. Was <u>automated</u> BP still elevated (≥ 90th percentile for patient's height, weight and sex, or ≥120/80)?

☐ No [**STOP COMPLETING FORM**]

☐ Yes (≥ 90th percentile for patient's height, weight and sex, or ≥120/80) [Go to 5]

4b. What were <u>manual</u> BP results: Systolic Blood Pressure: ______________
Diastolic Blood Pressure: ______________

4c. Was BP re-measured again <u>with manual</u> ?

☐ Yes [Go to 4c.i] ☐ No [Go to 4e]

4c.i. If Yes, what were <u>additional</u> results: Systolic Blood Pressure #2: ______________
Diastolic Blood Pressure #2: ______________ [Go to 4d]

4d. Was <u>average</u> of 2 <u>manual</u> BPs still elevated (≥ 90th percentile for patient's height, weight and sex, or ≥120/80)?

☐ Yes [Go to 5] ☐ No [**STOP COMPLETING FORM**]

4e. Was first <u>manual BP elevated</u> (≥ 90th percentile for patient's height, weight and sex, or ≥120/80)?

☐ Yes [Go to 5] ☐ No [**STOP COMPLETING FORM**]

5. Did provider document that the following were discussed with patient and/or family:

5a. Weight counseling ☐ Yes ☐ No ☐ N/a patient has normal BMI

5b. Nutrition counseling ☐ Yes ☐ No

5c. Lifestyle modifications ☐ Yes ☐ No

6. Did the patient have BP in Stage II Hypertension range (≥95th+12mm to <95th+30mm; or ≥140/90 to <180/120)?

☐ Yes [Go to 6a] ☐ No [Go to 7]

6a. Did the patient have BP in Stage II Hypertension range (≥95th+12mm to <95th+30mm; or ≥140/90 to <180/120) <u>at the prior visit</u>?

☐ Yes [Go to 6b] ☐ No [Go to 7]

6b. Did the provider document a diagnosis of 'Stage II Hypertension' in notes, billing or problem lists at this visit?

☐ Yes [Go to Section F] ☐ No [Go to Section F]

7. Did the patient have BP in Stage I Hypertension range (≥95th to <95th + 12mm or 130/80 to 139/89)?

☐ Yes [Go to 7a] ☐ No [Go to 8]

7a. Did the provider document a diagnosis of 'Stage I Hypertension' in notes, billing or problem lists at this visit?

☐ Yes [Go to Section F] ☐ No [Go to Section F]

8. Did the provider document a diagnosis of 'Elevated Blood Pressure (or BP)' in notes, billing or problem lists at this visit?

☐ Yes [Go to Section F] ☐ No [Go to Section F]

F. Actions Taken

1. What co-morbidities does the patient have or is suspected of having based on documentation in the chart?

☐ Diabetes ☐ Abnormal renal function (e.g. elevated Cr) ☐ Obesity/Overweight ☐ Obstructive Sleep Apnea

☐ Growth Delay ☐ Endocrine disease history ☐ Social concerns (e.g. drug use, school truancy, etc.)

☐ Other: ____

2. Were any relevant BP laboratory evaluations completed on this patient? ☐ Yes [Go to 3] ☐ No [Go to 5]

3. Date of first laboratory evaluations *resulted* (MM/DD/YYYY): _ _ /_ _ / _ _ _ _ [THESE DATA ARE ENTERED INTO REDCAP BUT NOT GIVEN TO RESEARCH TEAM]

*Not date laboratory evaluation ordered or sent, but date labs actually *resulted* back to clinic

4. Which laboratory evaluations have *resulted* since patient had 2nd or 3rd elevated BP readings (Check all that apply)?

☐ CBC ☐ Chemistry Panel (Cr, BUN, electrolytes) ☐ Urine Analysis ☐ Fasting Serum Glucose

☐ Lipids ☐ AST/ALT ☐ HgA1c ☐ TSH ☐ Drug screen

☐ Other: ____

5. Were any relevant radiologic or other evaluations *completed* on this patient? ☐ Yes [Go to 6] ☐ No [Go to 8]

6. Date of first radiologic or other evaluations *resulted* (MM/DD/YYYY): _ _ /_ _ / _ _ _ _ [THESE DATA ARE ENTERED INTO REDCAP BUT NOT GIVEN TO RESEARCH TEAM]

*Not date radiologic or other evaluations ordered or completed, but date actually *resulted* back to clinic

7. Which radiologic or other evaluations have resulted (Check all that apply)?

☐ Renal US ☐ Sleep Study ☐ CTA ☐ MRA

☐ Other: ____

8. If laboratory evaluations or radiologic/other evaluations were abnormal, was a referral request to a subspecialist made?

☐ Yes, abnormal results led to a subspecialist referral request [Go to 8a]

☐ No, abnormal results did not lead to a subspecialist referral request [Go to 9]

☐ No referral because all laboratory evaluations and radiologic/other evaluations normal [Go to 9]

☐ Referral request made to specialist but all laboratory evaluations and radiologic/other evaluations normal [Go to 8a]

8a. If yes, what date was referral request *made*? (MM/DD/YYYY): _ _ /_ _ / _ _ _ _ [THESE DATA ARE ENTERED INTO REDCAP BUT NOT GIVEN TO RESEARCH TEAM]

*Not date of subspecialist visit, what date did someone in the clinic tell the patient/family they needed to see a subspecialist and complete referral paperwork?

8b. If a subspecialist referral was made, which specialist? (Check all that apply):

☐Nephrologist ☐Cardiologist ☐ Other: ____

8c. ☐N/A: Patient never attended subspecialist visit [Go to 9]

[IF N/A NOT CHECKED] Date first subspecialist visit <u>completed</u> (MM/DD/YYYY): _ _ /_ _ / _ _ _ _ [THESE DATA ARE ENTERED INTO REDCAP BUT NOT GIVEN TO RESEARCH TEAM]

*<u>Not date referral request made</u>, but date patient actually saw subspecialist

☐

8d. Date information on the patient was received from subspecialist back to the pediatrician: (MM/DD/YYYY): _ _ /_ _ / _ _ _ _ [THESE DATA ARE ENTERED INTO REDCAP BUT NOT GIVEN TO RESEARCH TEAM]

*If same electronic health record, enter date note was signed by the subspecialist

9. Was an ambulatory blood pressure monitoring (ABPM) device used in this patient? ☐ Yes [Go to 9a] ☐ No [Go to 10]

9a. If Yes, who prescribed the ABPM? ☐ Pediatrician ☐Subspecialist ☐ Other: ______________________________

9b. How useful was the information from the ABPM?

☐ Very Useful ☐ Useful ☐ Neutral ☐Not Useful at All ☐Do Not Know

10. Were BP readings obtained in the school setting used in this patient? ☐ Yes [Go to 10a] ☐ No [Go to Section G]

10a. If Yes, who prescribed the school setting BPs? ☐ Pediatrician ☐Subspecialist ☐ Other: _________________________

10b. How useful were the school setting BPs?

☐ Very Useful ☐ Useful ☐ Neutral ☐Not Useful at All ☐Do Not Know

G. Pharmacologic Management

1. Was an echocardiogram completed on this patient? ☐ Yes [Go to 2] ☐ No [Go to 3]

2. Date of echocardiogram *resulted* (MM/DD/YYYY): _ _ /_ _ / _ _ _ _ [THESE DATA ARE ENTERED INTO REDCAP BUT NOT GIVEN TO RESEARCH TEAM]

*<u>Not date echocardiogram ordered or completed</u>, but date actually resulted back to clinic

2a. If echocardiogram was abnormal, was a referral to a subspecialist made?

☐ Yes, <u>abnormal</u> results led to a subspecialist referral [Go to 2b]

☐ No, <u>abnormal</u> results did not lead to s subspecialist referral [Go to 3]

☐ No referral because echocardiogram <u>normal</u> [Go to 3]

☐ Referral made to specialist but echocardiogram <u>normal</u> [Go to 2b]

☐ Patient already referred to subspecialist for echocardiogram [Go to 3]

2b. If yes, what date was referral request *made*? (MM/DD/YYYY): _ _ /_ _ / _ _ _ _ [THESE DATA ARE ENTERED INTO REDCAP BUT NOT GIVEN TO RESEARCH TEAM]

*<u>Not date of subspecialist visit</u>, what date did someone in the clinic tell the patient/family they needed to see a subspecialist and complete referral paperwork?

2c. If a subspecialist referral was made, which specialist? (Check all that apply):

☐Nephrologist ☐Cardiologist ☐ Other: ____

2d. ☐N/A: Patient never attended subspecialist visit [Go to 3]

[IF N/A NOT CHECKED] Date first subspecialist visit completed (MM/DD/YYYY): _ _ /_ _ / _ _ _ _ [THESE DATA ARE ENTERED INTO REDCAP BUT NOT GIVEN TO RESEARCH TEAM]

*Not date referral request made, but date patient actually saw subspecialist

2e. Date information on the patient was received from subspecialist back to the pediatrician: (MM/DD/YYYY): _ _ /_ _ / _ _ _ _ [THESE DATA ARE ENTERED INTO REDCAP BUT NOT GIVEN TO RESEARCH TEAM]

*If same electronic health record, enter date note was signed by the subspecialist

3. Was a medication to treat the patient's hypertension or elevated blood pressure started?

☐ Yes [Go to 4] ☐ No [**STOP COMPLETING FORM**]

4. Who started the medication?

☐Pediatrician ☐Nephrologist ☐Cardiologist ☐ Other: ____

5. Date medication ordered (MM/DD/YYYY): _ _ /_ _ / _ _ _ _ [THESE DATA ARE ENTERED INTO REDCAP BUT NOT GIVEN TO RESEARCH TEAM]

*Date medication ordered

Thank you for completing this data entry form

Recruitment Email for Specific Providers:

Dear Dr. XXX,

We reaching out to you as a leader of a pediatric primary care practice. As you know, between 3% and 5% of children have hypertension, and almost 40% of children diagnosed with hypertension already have lifelong heart muscle damage when they are diagnosed. Prior studies suggest pediatricians may be missing elevated blood pressure in their patients more than 80% of the time. For this reason, we are attempting to ensure accurate and timely pediatric hypertension diagnosis and management for all pediatric patients, based on the 2017 AAP guidelines. As part of this research study, we are also looking to better integrate subspecialist and primary care practitioners in this effort, and even move more primary care practitioners towards managing hypertension themselves with subspecialist back-up as needed. We are pursuing this effort led by a group of 10 nationally known pediatric quality improvement, primary care and nephrology researchers. Also, clinics from Johns Hopkins, University of Michigan, Cleveland Clinic, Montefiore Medical Group, and Medical University of South Carolina are already enrolled.

The study will randomize primary care clinics and their associated pediatric subspecialists (nephrologist and/or cardiologist) to one of 3 groups. All groups will get the same quality improvement collaborative intervention in a stepped wedge fashion. The first group will begin working on improving measurement of blood pressure, recognition of elevated blood pressure, and initial steps towards hypertension diagnosis and management. After 6 months, they will incorporate their subspecialist into the collaborative, to improve communication between the primary and subspecialty care realms. Following those 6 months, we will encourage primary care practices to move further with a “hub and spoke” model; their subspecialist will serve as a “hub” for hypertension diagnosis and management questions, to multiple primary care clinic “spokes” who will be doing more and more hypertension care themselves. We anticipate this will benefit subspecialists as they can see more new patients, benefit patients who will not need to travel to a tertiary care center and get more rapid care, and finally primary care physicians who can bill for higher acuity visits and have greater patient continuity. Group 1 will spend the final 6 months of the project sustaining these improvements and changes. Group 2 will proceed similarly through this list with a 6 month “usual care” with data collection phase in the beginning and no sustain phase at the end. Group 3 will have a 12 month usual care phase and not have the intermediate phase where primary and subspecialty care practitioners work on communication and transition issues. Practices will be involved for a total of approximately 29 months as some initial pre-work is needed. We anticipate the collaborative will begin pre-work in winter 2018-2019.

Each practice will be asked to identify and track the care delivered for approximately 10 patients with elevated blood pressure readings (not ‘Elevated Blood Pressure’ diagnosis) each month. They will also join every 6 month video conference day-long learning sessions when they are intervening, and every month 1-hour long collaborative video conferences. Everyone involved can get Part 4 MOC credit, one on one QI coaching, and we hope (but cannot promise until enrollment is complete) to be able to provide some small amount of money to each site to reduce data collection burden.

If this project still sounds interesting to you, we would first and foremost ask that you identify a primary care practitioner(s) and a pediatric nephrologist or cardiologist who will be your co-leads for this study. As much of this project is centered around the care delivered in primary care, the primary care co-leader will be essential to its success. We also **suggest that each subspecialist identify at least 2 (or more) clinics** who they can work with, as we want to make sure the subspecialist has enough hypertensive patient volume referred from the primary care clinics to make it worthwhile for them to participate.

Once you have your partner(s) and if you are still interested, please reach out to me. We can find days and times when the project leadership and your team can get on a conference call to discuss further specifics and answer any and all questions.

Thank you again for your interest and all the best,
--Michael

Michael L. Rinke, MD, PhD
Medical Director of Pediatric Quality
Associate Professor of Pediatrics
Attending Physician, Pediatric Hospital Medicine

The Children's Hospital at Montefiore
The Pediatric Hospital for Albert Einstein College of Medicine
3411 Wayne Avenue, Bronx, NY 10467
718-741-2597 Office
mrinke@montefiore.org
www.montekids.org

Quality Improvement Program Assistant
Peterkaye Kelly: pekelly@montefiore.org
718-741-2524

Recruitment Email for Pediatric Quality Improvement Listserves:

Dear XXX Members,

We are reaching out to you as leaders of pediatric primary care practices. As you know, between 3% and 5% of children have hypertension, and almost 40% of children diagnosed with hypertension already have lifelong heart muscle damage when they are diagnosed. Prior studies suggest pediatricians may be missing elevated blood pressure in their patients more than 80% of the time. **For this reason, we are attempting to ensure accurate and timely pediatric hypertension diagnosis and management for all pediatric patients, based on the 2017 AAP guidelines**. As part of this quality improvement and research study, we are also looking to better integrate subspecialist and primary care practitioners in this effort, and even move more primary care practitioners towards managing hypertension themselves with subspecialist back-up as needed. We are pursuing this effort led by a group of 10 nationally known pediatric quality improvement, primary care and nephrology researchers. Also, clinics from Johns Hopkins, University of Michigan, Cleveland Clinic, Montefiore Medical Group, and Medical University of South Carolina are already enrolled.

If you are interested in joining a pediatric quality improvement collaborative study to improve pediatric hypertension diagnosis and management, we would first and foremost ask that you identify a primary care practitioner(s) and a pediatric nephrologist or cardiologist who will be your co-leads for this study. As much of this project is centered around the care delivered in primary care, the primary care co-leader will be essential to its success. We also **suggest that each subspecialist identify at least 2 (or more) clinics** who they can work with, as we want to make sure the subspecialist has a large enough hypertensive patient volume referred from the primary care clinics to make it worthwhile for them to participate.

Once you have your partner(s), please reach out to me (mrinke@montefiore.org) or join one of our introductory webinars:

- Thursday August 30th 2-3PM EST
- Wednesday September 5th, 3-4PM EST
- Login information same for both dates:
  Join from PC, Mac, Linux, iOS or Android: https://einsteinmed.zoom.us/j/6453977588
  US: +1 646 558 8656 or +1 669 900 6833
  Meeting ID: 645 397 7588

Thank you again for your interest and all the best,
--Michael

Michael L. Rinke, MD, PhD
Medical Director of Pediatric Quality
Associate Professor of Pediatrics
Attending Physician, Pediatric Hospital Medicine

The Children's Hospital at Montefiore
The Pediatric Hospital for Albert Einstein College of Medicine
3411 Wayne Avenue, Bronx, NY 10467
718-741-2597 Office
mrinke@montefiore.org
www.montekids.org

Quality Improvement Program Assistant
Peterkaye Kelly: pekelly@montefiore.org
718-741-2524

The information contained in this message may be private and confidential, and may also be subject to the work product doctrine. This email may also be considered confidential and privileged in response to the Montefiore Quality Council under Section 2805-M of the Public Health Law for all quality improvement and peer review purposes. Any unauthorized review, use, disclosure or distribution is prohibited. If you are not the intended recipient, please contact me by reply email and destroy all copies of the original message.

BP-CATCH 2.0 CHART REVIEW TOOL v. 4 2/16/2021

Patient Information: **FOR CLINIC USE ONLY; NOT ENTERED INTO REDCap**

1. Patient Last Name: ___________________________
2. Patient First Name: ___________________________
3. MRN: _____________________________________
4. Date of Birth (MM/DD/YYYY): _ _ /_ _ / _ _ _ _
5. REDCap Record ID: ____

<u>EXCLUDE Patients</u>: with repeat blood pressure measurements visit since last BP-CATCH visit.

H. Office

1. How many attempts did the clinic make before getting in touch with the patient's caregiver (e.g. parent)?
   □ 1 □ 2 □ 3 □ 4 □ 5 □ 6
2. Was clinic able to get in touch with patient's caregiver (e.g. parent)? □ Yes □ No **IF NO, STOP COMPLETING FORM**
3. Was caregiver interested in bringing patient to clinic for repeat BP measurements?
   □ Yes □ No **IF NO, STOP COMPLETING FORM**
4. Date of Clinic Visit: _ _ /_ _ / _ _ _ _
5. Patient age (in years) at this visit: __________________ years (round to nearest 0.5 year)
6. Height **<u>at this visit with an elevated blood pressure</u>** _____ (centimeters) □Not done at this visit
7. Weight **<u>at this visit with an elevated blood pressure</u>** _____ (kilograms) □Not done at this visit

I. Office Manual Blood Pressure (BP) Measurements

1. **First manual BPs results**
   - 1a. Systolic Blood Pressure: _____
   - 1b. Diastolic Blood Pressure: _____
2. **Second manual BPs results**
   - 2a. Systolic Blood Pressure: _____
   - 2b. Diastolic Blood Pressure: _____
3. Was teaching on Home Blood Pressure Monitor (HBPM) done by Clinic?
   □ Yes □ No

J. Home Blood Pressure Measurements

1. Date Patient received HBPM (MM/DD/YYYY): _ _ /_ _ / _ _ _ _
2. Date Patient started using HBPM (MM/DD/YYYY): _ _ /_ _ / _ _ _ _

		**BP Reading # 1 (mmHg)**		**BP Reading # 2 (mmHg)**		**BP Reading # 3 (mmHg)**	
	**<u>Date</u>**	*Systolic*	*Diastolic*	*Systolic*	*Diastolic*	*Systolic*	*Diastolic*
**<u>Day 1 Morning</u>**	/ /						
**<u>Day 1 Evening</u>**	/ /						
**<u>Day 2 Morning</u>**	/ /						
**<u>Day 2 Evening</u>**	/ /						

**Day 3 Morning**	/ /						
**Day 3 Evening**	/ /						
**Day 4 Morning**	/ /						
**Day 4 Evening**	/ /						
**Day 5 Morning**	/ /						
**Day 5 Evening**	/ /						
**Day 6 Morning**	/ /						
**Day 6 Evening**	/ /						
**Day 7 Morning**	/ /						
**Day 7 Evening**	/ /						

4. Did the Clinic received the HBPM data from Patient?

□ Yes □ No

4a. If yes to the above question, what date did the Clinic received the HBPM data? _ _ /_ _ / _ _ _ _

5. Was Provider notified of HBPM readings?

□ Yes □ No

K. Ambulatory Blood Pressure Monitor (ABPM)

1. Based on HBPM and Manual BP Measurements, did the patient meet the criteria to receive an ABPM and receive a nephrology referral? *ABPM given if patient had discordant hypertension stages (e.g. normal, elevated, Stage 1 or Stage 2) between HBPM and manual BP measurements and 8 or older, all discordant patients receive nephrology referral

□ Yes □ No **[STOP COMPLETING FORM]**

2. Was caregiver interested in receiving an ABPM and nephrology referral?

□ Yes □ No **IF NO, STOP COMPLETING FORM**

3. Date Patient picked up ABPM (MM/DD/YYYY): _ _ /_ _ / _ _ _ _ or □ N/A because patient <8 years old

4. Date Patient came for nephrology referral (MM/DD/YYYY): _ _ /_ _ /

or □ Not interested in nephrology referral □ Did not need nephrology referral

5. Date Patient started ABPM (MM/DD/YYYY): _ _ /_ _ / _ _ _ _

6. Date Patient returned ABPM (MM/DD/YYYY): _ _ /_ _ / _ _ _ _

7. Date ABPM summary page(s) scanned and emailed to PK and Provider: _ _ /_ _ / _ _ _ _

8. Final diagnosis per ABPM and nephrology: ___________________________

Thank you for completing this data entry form
