# Supplementary material for: Racial and Ethnic Disparities in Pediatric Counseling on Nutrition, Lifestyle, and Weight: A Secondary Analysis of the BP-CATCH Randomized Clinical Trial
Source: JAMA Netw Open. 2025 Jan 29;8(1):e2456238. doi: 10.1001/jamanetworkopen.2024.56238 (PMC11780477; doi:10.1001/jamanetworkopen.2024.56238)
Supplement: Supplement 2. — eFigure. CONSORT diagram [file jamanetwopen-e2456238-s002.pdf]

Supplemental Online Content

Heo M, Rea CJ, Brady TM, et al. Racial and ethnic disparities in pediatric counseling on nutrition, lifestyle, and weight. *JAMA Netw Open.* 2025;8(1):e2456238. doi:10.1001/jamanetworkopen.2024.56238

eFigure. CONSORT diagram

This supplemental material has been provided by the authors to give readers additional information about their work.

© 2024 Heo M et al. *JAMA Network Open*.

Supplementary Figure 1: BP-CATCH CONSORT Flow Chart Diagram

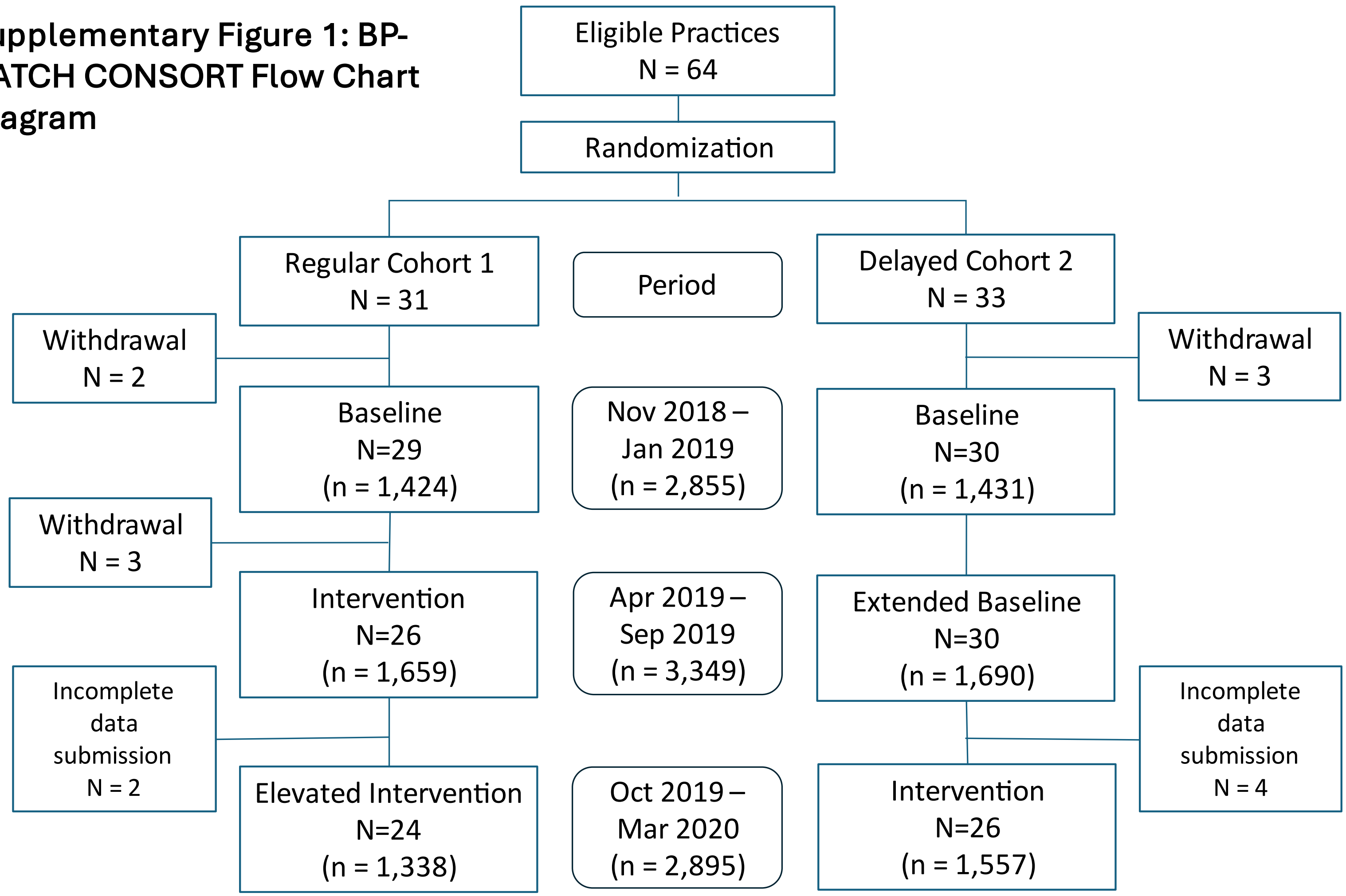

N: Number of practices; n: number of reviewed medical charts/visits

© 2024 Heo M et al. *JAMA Network Open*.
