# Supplementary material for: Racial and Ethnic Disparities in Pediatric Counseling on Nutrition, Lifestyle, and Weight: A Secondary Analysis of the BP-CATCH Randomized Clinical Trial
Source: JAMA Netw Open. 2025 Jan 29;8(1):e2456238. doi: 10.1001/jamanetworkopen.2024.56238 (PMC11780477; doi:10.1001/jamanetworkopen.2024.56238)
Supplement: Supplement 3. — Data Sharing Statement [file jamanetwopen-e2456238-s003.pdf]

Data Sharing Statement

Heo. Racial and Ethnic Disparities in Pediatric Counseling on Nutrition, Lifestyle, and Weight. *JAMA Netw Open*. Published January 29, 2025. doi:10.1001/jamanetworkopen.2024.56238

Data
Additional Information: This study was registered on ClinicalTrials.gov as Boosting Primary Care Awareness and Treatment of Childhood Hypertension (BP-CATCH), NCT03783650.
Data available: No

Additional Information
Explanation for why data not available: The raw data are currently unavailable to the public.
